# Training in the art and science of facilitation to scale research mentor training in low and middle income countries

**DOI:** 10.3389/feduc.2023.1270480

**Published:** 2023-12-21

**Authors:** Bennett B. Goldberg, Erasto V. Mbugi, Fatima Kyari, Sara E. Woods, Emmanuel Balandya, Denise Drane, Rifkatu Reng, Deodatus Kakoko

**Affiliations:** 1 Department of Physics and Astronomy, Northwestern University, Evanston, IL, United States,; 2 Department of Biochemistry and Molecular Biology, School of Biomedical Sciences, College of Medicine, Muhimbili University of Health and Allied Sciences, Dar es Salaam, Tanzania,; 3 Department of Ophthalmology, University of Abuja, Abuja, Nigeria,; 4 Program Evaluation Core, Northwestern University, Evanston, IL, United States,; 5 Department of Physiology, School of Biomedical Sciences, College of Medicine, Muhimbili University of Health and Allied Sciences, Dar es Salaam, Tanzania,; 6 Department of Internal Medicine, University of Abuja, Abuja, Nigeria,; 7 Department of Behavioural Sciences, School of Public Health and Social Sciences, Muhimbili University of Health and Allied Sciences, Dar es Salaam, Tanzania

**Keywords:** facilitation, mentoring, mentor training courses, low and mid income countries, equity and social justice

## Abstract

Advancing biomedical research in low and middle income countries (LMICs) to expand the capacity for LMICs to integrate biomedical research into their health care systems and education has been the focus of many programs in global health over the past two decades. Central to the success of these programs is effective research mentoring, characterized by academic, career and psychosocial support through culturally appropriate practices. Research mentoring is a learned skill, developed through training, mutual discussions, practice and feedback. The majority of extant training programs are designed and delivered by US partners, so the next stage in building capacity is to train facilitators within the LMIC partner institutions to contextualize and advance mentoring specifically within their cultural and institutional norms by co-developing, delivering and evaluating semi-annual research mentoring training. To this end, we describe the development, delivery and outcome evaluation of a 5-week course in the art and skill of facilitation. Care was taken to explicitly distinguish between concepts of “teaching” and “facilitation,” since “teaching” is closely connected to a transmission or banking model of education, which is characterized by “top-down,” hierarchical relationship. The course discussed power and positionality, themes that resonate with partners in Nigeria and Tanzania. These themes provided unique entry into deeper conversations core to advancing mentoring practice away from the traditional dyadic power structure that remains from colonization. Evaluation findings indicate significant advances in awareness of differences between teaching and facilitating, increased confidence in facilitation skills, especially in the area of structured planning and organization, as well as improved communication and interpersonal skills. All respondents felt that students in Nigeria and Tanzania would respond well to the facilitation approach conveyed during the course and they found value in participating in the course as a cohort.

## 1 Introduction: background and rationale for the educational activity innovation

The successful advancement of biomedical research in low-and middle-income countries (LMICs) requires a combination of education, infrastructure, and opportunity that combine to develop both the research and professional skills of biomedical researchers (Heimburger et al., 2016; Zunt et al. 2016; Franzen and Chandler, 2017). For two decades, programs like Fogarty Global Health Program for Fellows and Scholars (Zunt et al., 2016) have built research capacity in LMICs, with a focus on training to advance a country’s abilities to successfully address their health challenges, develop a cohort of local biomedical researchers, build infrastructure and implement a robust medical education system to self-sustain and grow (Potter and Brough, 2004; Cooke, 2005; Hargreaves et al., 2019; Lescano et al., 2019).

Professional skills such as mentoring, leadership, and communication, though generally considered less critical than research skills, are necessary supporting elements of research and academic success (Pfund et al., 2016), especially in academic training environments. Nurturing trainees who are engaged in local and collaborative research between LMICs and developed countries is critical to the success of the research and to trainee development. Mentorship builds capacity for research by facilitating the entry of developing researchers into the community of practice (National Academies of Sciences, Engineering, and Medicine, 2019), by harmonizing a mesh of experiences into common values and structures and thus provides institutional research growth and sustainability. The World Health Organization (Health research mentorship in low and middle-income countries (HERMES), 2022) has further reiterated research mentorship to be a powerful fundamental tool in science by not only leveraging recognized expertise to strengthen individual scientists and institutional capacity, but by helping to mold generations of researchers with positive influence on their career development by instilling a learning culture in research. Research mentorship is a core capacity in the process of generating research knowledge, communicating findings, and addressing important aspects of equity in health and education.

Effective research mentoring is characterized by academic, career and psychosocial support through culturally aware practices that further the careers of trainees and early career scientists (Pfund et al., 2016; Noormahomed et al., 2019; Womack et al., 2020; Byars-Winston et al., 2023). Mentoring is a learned skill, developed through training, mutual discussions, practice and feedback (Hokanson and Goldberg, 2018). Over the past decade, research mentor training has grown from an area of relative weakness (Cole et al., 2016) to an area which has demonstrated more widespread implementation, changing both practice and perspectives (Pfund et al., 2014; Gandhi et al., 2019; Hamer et al., 2019; Desai et al., 2021; Alidina et al., 2022; Sun, T., et al., 2023). Yet, mentorship in LMICs remains challenged by few training hubs and platforms, poor institutionalization, turnover and loss of expertise, and limited recognition and funding [Hansoti et al., 2019; Oppong et al., 2021; Health research mentorship in low and middle-income countries (HERMES), 2022].

In our own practice, we co-developed and co-implemented three, two-day research mentoring workshops in Nigeria and Tanzania, exploring multiple mentoring domains, and co-constructing a triad model with an indigenous mentor and a US-based mentor collaborating in support of a trainee. The workshops sought to shift mentoring practice and perspectives to trainee-focused, and away from supervisory roles. It is important to note that almost all mentoring training programs in LMICs, like ours, are exclusively developed and implemented by researchers from the U.S. (Rose et al., 2022;Deprez et al., 2023; Rose et al., 2023).

Mentor training in the U.S. has seen a large recent growth, due in part to a shift from a limited number of experts delivering workshops to scaling and implementing nationwide train-the-facilitator programs that directly address the most significant bottleneck to widespread mentoring practice, the lack of skilled facilitators (Pfund et al., 2017; Rogers et al., 2018; Spencer, 2018). These studies demonstrate the successful expansion and high fidelity of train-the-facilitator models. While training of facilitators to deliver local training is growing in access and implementation in the U.S., building this capacity in LMICs has not yet begun, and is an important need to support research capacity and academic advancement.

The art and science of facilitation requires both content knowledge and facilitation expertise. Facilitation is a learned skill and is different from teaching. Research on teaching practice has identified two broad categories, the transmission or banking model (Freire and Ramos, 1970) where the instructor is transferring their knowledge, generally through lecture, to the open vessel that is the student’s mind, and student-centered teaching or social constructivism, where the instructor guides the student to engage in questioning, reflection, and discourse that allows them to build knowledge for themselves (Dewey, 1938; Light et al., 2009). While facilitation is akin to a learner-centered instruction practice, it has fundamental differences. First, teaching, especially in a biomedical academic setting, seeks to transition the student from a novice to expert by gaining a range of competencies in the subject. There is typically a well-defined set of abilities and knowledge that the expert attains, defined as “correct.” Hence the instructor has a particular end goal in mind for the learner. Facilitation also guides the learner in constructing their own knowledge, but unlike teaching, intends the learner to contextualize and adapt the ideas, molding the content significantly to their own experience and needs. Facilitation’s goal is not well-defined as in teaching, it is more the development of one’s own perspectives and approaches that utilize effective, common structures, but do not necessarily reproduce them.

Training in facilitation, while different from training in teaching, supports instructors’ advancement in both the workshop and classroom learning environments. This is especially true in low-and middle-income countries whose educational systems still reflect the vestiges of colonialism (Kay and Nystrom, 1971). Western educational systems were imposed, bringing with them a hierarchical, banking model of teaching that reflected the power and positionality of the oppressors, the occupying colonial power. While some systems have thrown off those bonds, many have not (Wandela and Eugenia, 2014). For example, while some institutions have now revised their curricula from knowledge-based to competency-based approaches, the guidance by instructors might remain a “church worship” one where only the preacher remains the speaker due to the accustomed system of education adopted from colonial mode. Colonial systems of education changed the pre-colonial era system of education from progressive to essentialist education to enable achievement of the goal of colonization (Garba, 2012). Consequently, this approach remained in the minds of people post-colonialism as thus the facilitative mentorship training has become necessary. Facilitation provides the opportunity to transform from single, unidirectional mode in teaching to participatory mode where each key player has a chance to contribute his/her thoughts to the common goal. Teaching practice tends to reproduce transmission and lecturing, treating the students as receptacles of the instructor’s expertise, rather than the creator of knowledge. Active learning and learner-centered instruction are not yet common in Nigeria and Tanzania (Bonwell and Eison, 1991), as they are frankly not yet common in the west (Freeman et al., 2014; Dancy et al., 2022; Handelsman et al., 2022). Hence our project needed to develop facilitation professional development for academic medical faculty that acknowledged where they were in their own instructional practice.

Based on a continuing clear need for research mentoring in low-and middle-income countries in the health profession, the clarity that research capacity building has focused largely on research skills and competencies, and the challenge of addressing the bottleneck of a lack of trained facilitators to lead local workshops, we designed, implemented and evaluated the effectiveness of a five-week, synchronous, virtual course on the art and practice of facilitation. We describe the pedagogical approach and frameworks, the workshop design and environment, and the post-workshop evaluation and interviews. As part of our process, we are including the experiences and voices of participants, applying an autoethnographic approach in describing the work (Ellis et al., 2010).

## 2 Pedagogical frameworks, principles, and innovations underlying the educational activity

**FIGURE 1:**
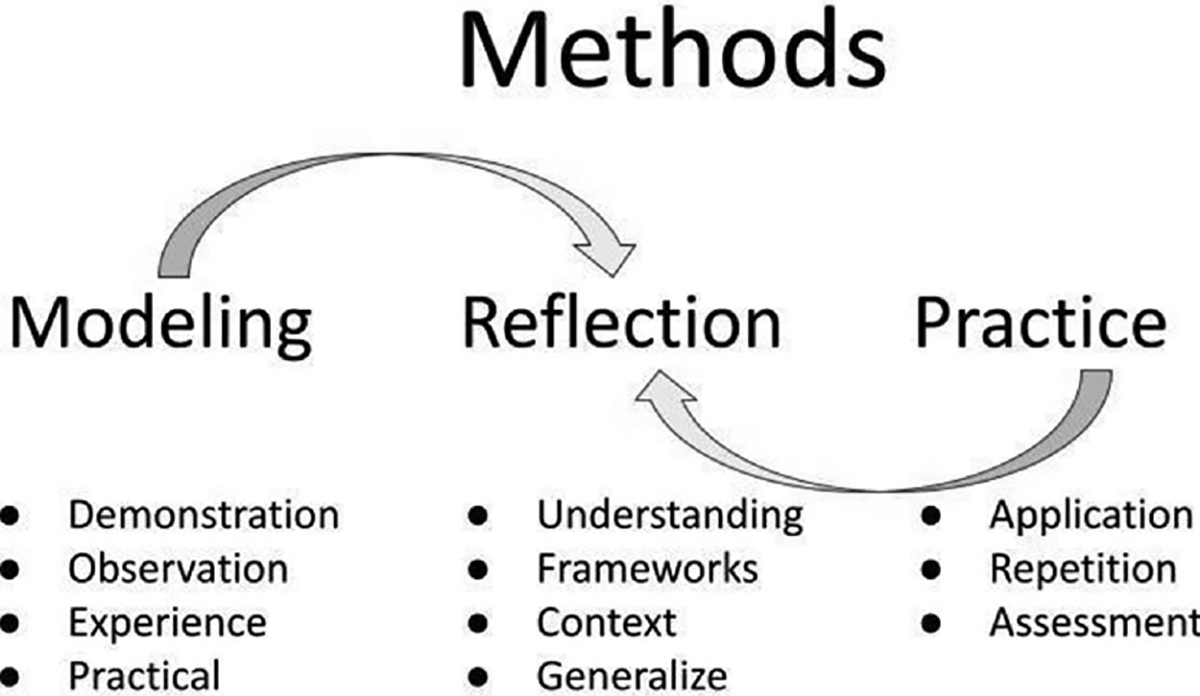
The pedagogical approach combined modeling with participant practice, coming together in reflection to develop learning and knowledge.

The pedagogical framework for the facilitation workshop was to engage participants directly in the practice of facilitation through modeling, reflection and practice. Figure 1 displays the central process of the approach. This framework models approaches to guide rather than lead discussions to advance learner agency. The approach is based in part on large-scale facilitation training models (Pfund et al., 2017; Rogers et al., 2018; Spencer, 2018) around research mentor training with the addition of multipartial facilitation (Zappella, 2007; Giacomini and Schrage, 2009; Routenberg et al., 2013) and an intentional focus on power, privilege and positionality in local mentoring contexts. The lead trainer (Goldberg) would model facilitation multiple times during a workshop, each time engaging the trainees in a group learning activity, leading co-creation and group work, followed by reflection of both the learned content, and in a metacognitive dimension, discussing how the activity was facilitated and how a trainee would do so in their own context. Through modeling, the trainees could observe and experience multiple modes of facilitation; through practice, the trainees were able to translate to their own perspective and literally “do” the facilitation; and through reflection, both trainer and trainee were able to collectively capture key elements and build mental models.

In addition, each day led with a core principle, that “we learn together.” Specifically, that (1) we are here together in the same rooms to learn from each other. The wisdom is in the room; (2) we seek to explore new ideas, practice together, role play, in the process of developing facilitation skills; and (3) we are aware of and acknowledge the power and positionality differential, and seek that such differential is, when necessary, overcome to enhance learning.

Finally, many elements of the workshop focused on creating and sustaining learner agency, participation, and co-creation, all hallmarks of active learning strategies (Brame, 2016). These techniques were translated to the online format and were equally effective as in-person active learning approaches (Goldberg, et al., 2023). Examples include multiple breakout sessions with directed activities, specified roles (Brame, 2016), and group report; regular use of shared documents and creative spaces with Padlets, Google Docs, Jamboards, and chat streams; and pre-session and post-session asynchronous learning and engagement.

## 3 Learning environment, learning objectives, and pedagogical format

Due to the global COVID pandemic, the learning environment was an online format. Five 90-min sessions were held once per week between the beginning of August and early September, 2022. Pre-session materials included readings, reflection and an occasional video. Post-session included recap and reflection, completion of synchronous activities, and access to additional materials. This format was successful in balancing the time available and busy schedules of the participants - university faculty and administrators - with the ability to have some synchronous and asynchronous learning spaces.

The participants in the facilitation workshops were medical and nursing school faculty in clinical positions at Muhimbili University of Health and Allied Sciences (MUHAS), Dar es Salaam, Tanzania and the University of Abuja, Abuja, Nigeria. Four of the participants hold multiple titles, including that of Director or Dean at their respective schools. Most of the faculty are at a mid-career to senior level in their department, either as a lecturer and/or clinician and researcher. Nine participants have been teaching for 10 years or more. Eight of the faculty members were male and five were female. The participants were invited as part of two National Institutes of Health Fogarty D43 biomedical research capacity building projects, one at MUHAS focused on patient-centered research outcomes and one at the University of Abuja focused on cardiovascular research. Both cites had received prior research mentor training workshops, described above, and both sites sought to expand their capacity to lead their own research mentoring workshops.

The overall program learning goals were for participants in the series to

1. Describe the foundational elements of effective facilitation, the key features of active learning and high engagement, and apply the principles of backward design to create learner-centered content and experiences.
2. Develop and produce small group learning interactions; be able to describe the key steps and processes, identify facilitation challenges and opportunities, and support multiple interactions.
3. Identify appropriate areas of assessment and evaluation of effective facilitation and use these in an observation rubric; be able to produce and perform one’s own designed small group learning interaction; be able to learn from peer-and expert evaluation.

Session 1: The learning objectives of session one were for learners to meet each other and build trust through asset declarations, reflect on their own expectations for the training, share their preconceptions of facilitation and the differences between facilitation and teaching, and co-created guidelines for discussion. The session introduced through modeling several key facilitation practices, including carefully directed breakouts with shared documents for collecting work product, and what should a facilitator first do when asked a question. This session built the foundation through presenting a social constructivist theory of learning and describing the three central modes of teaching and of learning (all session slides are included for reference in the Supplementary material).

**FIGURE 2:**
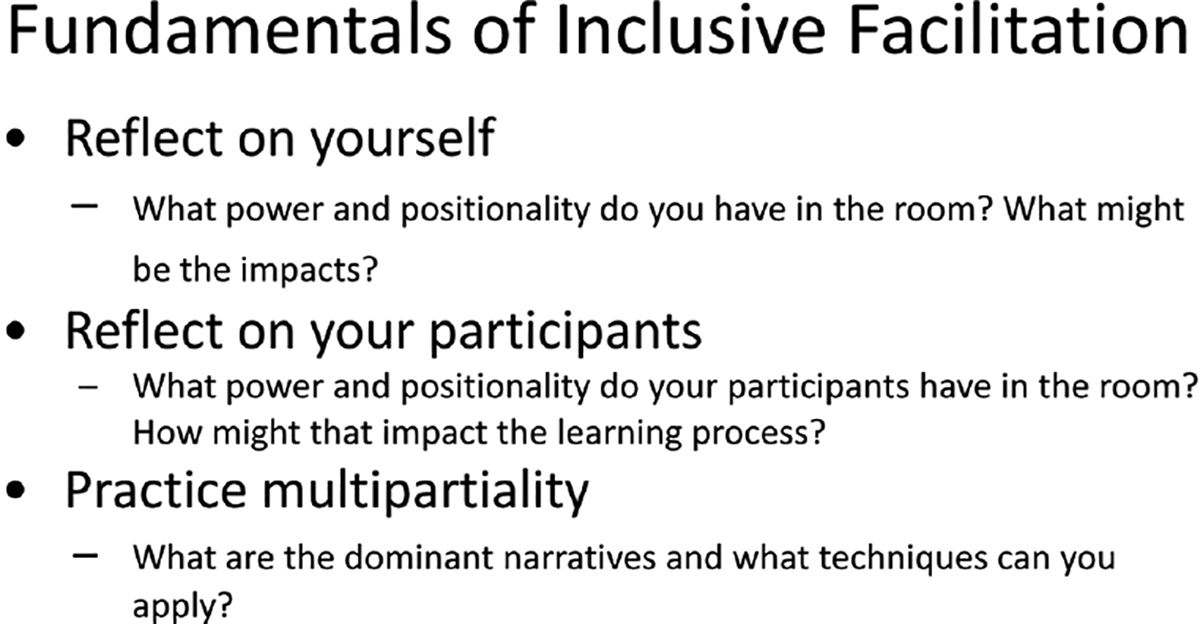
Fundamentals of inclusion facilitation discussed as part of the course, “Arts and Science of Facilitation”.

Session 2: The learning objectives of session two were for learners to continue to develop strong, collaborative and supportive relationships, explore pre-session assigned work around active learning, collaborative learning and peer instruction, and delve deeply into power and positionality and culturally aware multipartial facilitation practices (Routenberg et al., 2013). Active learning exercises were followed by a focus on fundamentals of inclusive facilitation displayed in Figure 2. These discussions centered the ideas of power and positionality, and participants were able to identify different aspects of gender, age, career status, and ethnic groups that impact learning, and how multipartiality can identify majority narratives to highlight and make space for minority narratives.

Session 3: The learning objectives of session three were to recap power, positionality and mulitpartial facilitation to examine inclusive facilitation practices and explore examples, to begin the process of workshop and run-of-show design using basic backward design principles of Wiggins and McTighe (2005). Backward design was connected to Bloom’s taxonomy, revised toward a cognitive framework (Anderson and Krathwohl, 2001) to emphasize the need for scaffolding the cognitive complexity of learning objectives and learning activities to reach deep learning goals. Participants began work on their own workshop design, and prior to sharing their ideas, engaged in a structured conversation about how to give effective, professional, and supportive feedback -- another key facilitation skill.

Session 4: The learning objectives of session four were to build out the backward design worksheet for their practice workshop, give and receive feedback in a structured mode, and examine the principles of a run-of-show design to guide the flow of the workshop. Participants shared their work and received structured feedback in small groups, followed by a meta cognitive reflection on the process itself and how it relates to facilitation. Key to facilitation success is a combination of content and pedagogy, which was particularly emphasized in this session.

Session 5: The learning objectives of session five were to explore and complete a run-of-show template See Supplementary materials, practice small-group facilitation, and consolidate the learning from the entire course through recap and reflection. In addition, as an action-reflection exercise, participants were asked questions about their own practice and described the spaces they were planning to use what they learned. Session five finished the run-of-show worksheets combined with the backward design workshop structure to provide participants with a complete sequence of developing an inclusive facilitation workshop on mentoring.

Completion certificates were awarded to participants who attended at least four of the five sessions. Twelve received certificates out of the initial 18 participants.

## 4 Results and outcomes

The program was evaluated by an evaluation specialist from Northwestern University’s Program Evaluation Core (2023). To guide the evaluation of this facilitation workshop series, we relied on the Kirkpatrick framework (Kirkpatrick, 1994, 1996) for evaluating training programs. Both in our post-workshop survey and one-on-one interviews, we focused mainly on measuring participant reaction to the training (level one of the framework) and the learning that took place as a result of the training (level two). Since we felt it was important to get immediate feedback from the participants, we only asked questions about *anticipated* changes in behavior (level three). We have not yet conducted a follow-up survey to measure the actual behavior change or the results of their changed behaviors (level four).

The goals for evaluation of the workshop series included:

1. Understand the extent to which and limitations of how the training engendered the participant’s ability to engage, absorb, and build facilitation perspectives and skills.
2. Create a clear image of the participant’s knowledge and skill gains, especially their own shifts in perspectives on teaching and facilitation.
3. Understand the change in participant’s behaviors as a teacher and facilitator. Explore the potential impacts of participant’s changed behaviors as a teacher and facilitator.

To holistically evaluate the workshop, we created a survey for participants to complete after the workshop series and an interview protocol. This mixed methods approach allowed for collection of quantitative, as well as qualitative data. Similar to many other post-training workshop surveys, this survey included questions about key outcomes relative to learning goals, described above. The survey questions were not validated as this study was a pilot and we did not have a large enough sample size to perform a validation study. Some of the questions from the survey were drawn from a validated survey. The survey included questions that required ranking on a Likert scale, as well as questions that solicited open-ended responses. A block of questions in the survey were drawn from The Critical Incident Questionnaire (CIQ; Brookfield, 1995, 2003), which is a tool for understanding classroom dynamics. Essentially, it allows the instructor to “see” the classroom through the perspective of the student (s). We chose to include this tool since it explores engagement as a proxy for learning within the activities of the workshop.

We did not seek IRB for this study because the study is considered to be an evaluation of an education program. Our institution’s Institutional Review Board does not consider this to be Human Subjects Research as we are evaluating outcomes of a specific educational program and not seeking to generate generalizable knowledge that extends beyond this program.

The survey was sent electronically at the close of the workshop. Thirteen of the eighteen workshop participants completed the survey for a 72% response rate.

Five of the 18 workshop participants agreed to participate in the interview portion of the evaluation. The main goal of the interview was to understand the experience of the workshop participants in their own words and in a deeper way than the quantitative data provided.

## 5 Evaluation findings

### 5.1 Surveys

The post-workshop survey was completed by 13 of the workshop participants. Results of the qualitative section of the survey (see Supplementary materials) indicated that the structure of the workshop was key for learner engagement. The majority of respondents said they were most engaged during the small group breakout portions of the workshop. Reasons given for the high level of engagement included ability to share knowledge, experiences and ideas with peers, opportunity to think critically and being in a small group encouraged a higher level of engagement in the activities.

The facilitation practices that participants saw as the most influential takeaways from the training included: learner-centered methods, giving everyone a voice/chance to talk, listening/being comfortable with silence and understanding the role of the facilitator. Participants were also asked which practices they were most likely to employ as a result of the training. The top practices included: facilitating open group discussions by giving learners the opportunity to express their ideas and share experiences, facilitating small group work and using a backward design approach.

**FIGURE 3:**
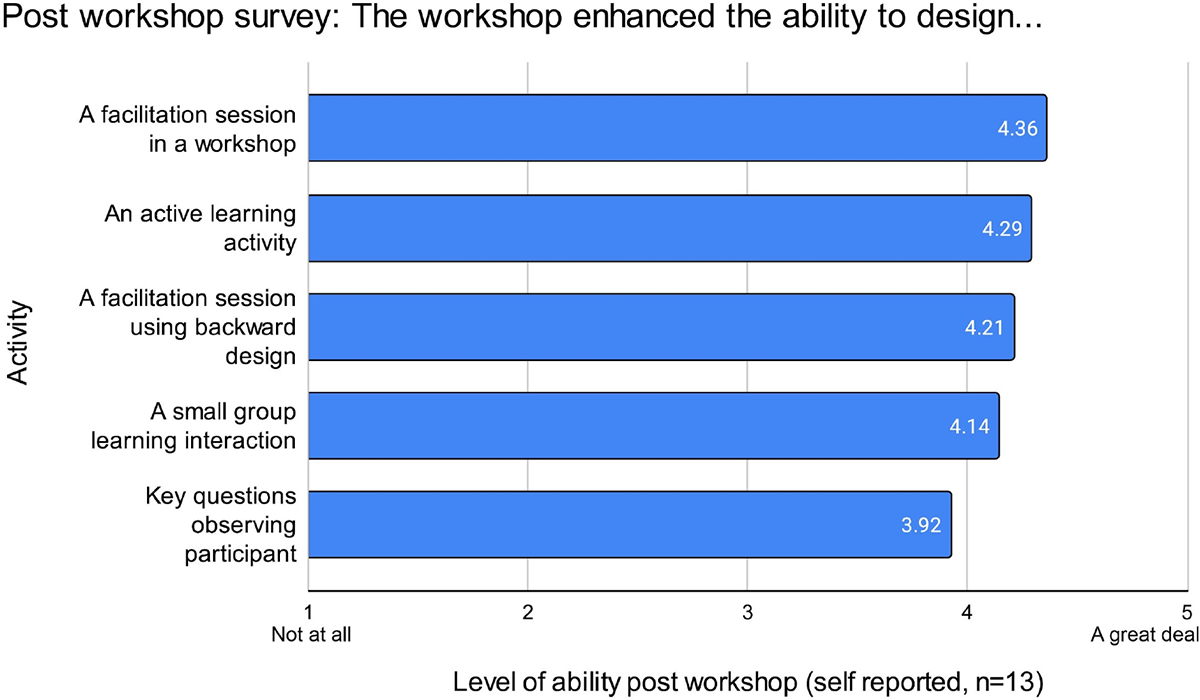
Post-workshop survey responses (*n* = 13) to the question. “On a scale of 1 to 5, where 1 is a little and 5 is a great deal, to what extent did the workshop enhance your abilities?” Note an overall high expression, with greatest enhancement of ability in facilitating a workshop.

The quantitative section of the survey (survey reproduced in the Supplementary material) asked a series of questions that requested the respondent to choose a Likert scale rating (the scale rating choices ranged from 1 = none at all to 5 = a great deal). Mean scores for the questions below are reflected in Figure 3.

- To what extent did the training advance your ability to design a facilitation session in a workshop?
- To what extent did the training advance your ability to design an active learning activity?
- To what extent did the training advance your ability to design a facilitation session using backward design?
- To what extent did the training advance your ability to design a small group learning interaction?
- To what extent did the training advance your ability to design a list of key questions for observing participant interactions in groups?

The post-survey responses indicate the training most advanced participants’ awareness of observing participant interactions in groups (mean 4.46, SD 0.63). The training also advanced participants’ ability to design a facilitation session in a workshop (mean 4.36, SD 0.61). Furthermore, all participants reported a confidence gain of greater than 3.0 in all areas measured which included facilitating small group learning, instructing active learning, using backward design, observing learners and shifting approach based on observation (see Figure 3).

The results revealed that participants felt the training advanced their knowledge across a wide range of key design skills from asking key questions around observing participant interactions to using backward design to designing a facilitation workshop overall. See Figure 3.

**FIGURE 4:**
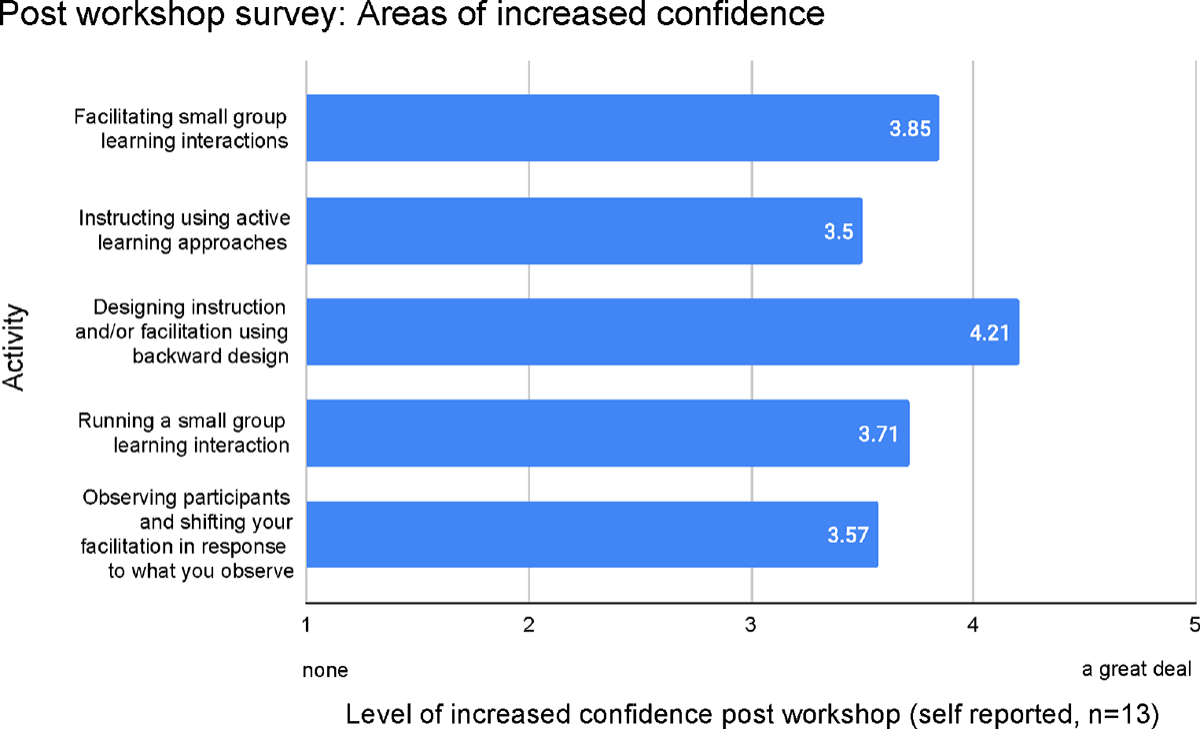
As indicated by post-workshop survey (*n* = 13) designing instruction and/or facilitation using backward design was the area of respondents reported the highest level of increased confidence.

The mean scores for the questions below are reflected in Figure 4.

- How much confidence have you gained in facilitating small group learning interactions?
- How much confidence have you gained in instructing using active learning approaches?
- How much confidence have you gained in designing instruction and/or facilitation using backward design?
- How much confidence have you gained in running a small group learning interactions?
- How much confidence have you gained in observing participants and shifting your facilitation in response to what you observe?

In terms of changes in confidence (Figure 4), we again observed large gains across the workshop learning goals, and in particular, confidence in designing instruction and/or facilitation using backward design, in alignment with enhanced ability. A notable area of significant increase was facilitating a small group learning interaction. The Likert scale used was 1 = none at all, 2 = a little, 3 = a moderate amount, 4 = a lot, 5 = a great deal.

### 5.2 Interviews

The interview protocol (see Supplementary materials) was built by the workshop facilitator and a team of two evaluators. Interview participants were asked about their levels of confidence, engagement and how they planned to use what they learned in the workshop. Five interviews took place over Zoom during fall 2022 about a month after the workshop sessions were complete. The interviews were audio recorded and transcribed. The same evaluation specialist facilitated the five interviews and took notes during each session and extracted themes from the interviews.

The interviewer created coding categories for each question and grouped the individual responses when they were similar in nature. For example, when asked the question, “Teaching means different things to different people, what does it mean to you?,” the coding categories were (1) imparting knowledge (2) to evoke change, and (3) transmission of information and abilities. For that question, three participants used “imparting knowledge” in their responses, while one participant spoke of evoking change and one participant defined teaching as a transmission of information and abilities.

All five interview participants were all able to quickly articulate the commonalities and differences between teaching and facilitation. They were asked, “Teaching means different things to different people, what does it mean to you?,” “What are the commonalities and differences between teaching and facilitation?” and “What are the commonalities and differences between facilitation and learning?” (The full interview protocol is available in the Supplementary material section). All respondents alluded to the fact that teaching and facilitation both involve imparting knowledge. One participant described the following when asked the differences between teaching and facilitation:

“Teaching is when someone is a novice, let them know some things, look at curriculum and deliver the knowledge. Facilitation is more active participation of the trainee, more interaction, gauge what they know and build upon that, increasing capacity to learn that thing.”

One respondent described the relationship between teaching and facilitation as such:

“Facilitation supports learning. Learning is a behavioral change that happens when facilitation is taking place.”

All interviewees reported an increased confidence in and awareness of facilitation practices. Specifically, several respondents became more aware of facilitation as a two-way process between facilitator and trainee. Other respondents said they had been using some of the practices, but did not know the proper names. For many, the workshop clarified the difference between teaching and facilitation.

Interviewees were asked what particular skills they had gained during the workshop. Responses included organizational skills, like building an agenda, run of show, managing course content and backward mapping. Other respondents reported an increase in knowledge around how to interact with and engage learners during a workshop.

Although the workshop approach was based on and developed in Western models of higher education, all participants felt that the approach is easily adaptable for their cultural context.

“Well, I think they’re picking up because, um, the youth these days, you know, are actually more vocal. I know that. And sometimes they come in a class expecting you to just teach them, to give them information, but now that they know that they are supposed to come up with what they can do, what they should say. They should feel free to say things.”

Workshop participants are planning to use their facilitation skills in a variety of contexts; in undergraduate classrooms, with medical school residents, in clinical training and during professional development workshops.

The training combined faculty from Tanzania and Nigeria. All participants reacted positively to learning with their peers from Nigeria and Tanzania when asked the question What was it like for you to work with faculty from Tanzania? (or Nigeria)? Several interviewees commented that teaching is universal, so the subject matter wasn’t a barrier to interaction. In fact, the small group breakouts made that interaction much easier, as noted by one participant:

“These things, you know, they are universal except that they are done different environments. So, it was not very difficult to start interacting as soon as we were split into groups. So, it was easier to initiate the discussion and then to everyone to contribute what or she or he knows and discuss and reach a conclusion, and then go back and forth and get feedback.”

The small group discussions provided opportunity for feedback, which the participants highly valued. Overall, the group appreciated the different backgrounds and perspectives of their peers, which led to a more enriching experience in the workshops. The majority of respondents would have liked more workshop sessions or longer sessions and one interviewee felt that more participants in the workshop would have also added value.

## 6 Autoethnographic reflections

Autoethnography was used to capture participant experience and impact of the workshop. Autoethnography is a technique that asks participants to share as authors, describe in their own words their experiences, and thus bring forward a more direct and meaningful experience and deeper cultural meaning (Ellis et al., 2010). In the following sections, we include the direct words of three participants to challenge the sole reliance on traditional means of doing research that represents the voices and thoughts of others. In this way, we balance the quantitative and qualitative evaluation described above with direct descriptions of participants.

Deodatus Kakoko, Associate Professor of Public Health, Department of Behavioral Sciences, Director of Continuing Education and Professional Development, MUHAS, Tanzania.

I started reflecting on facilitation immediately in the first session as the facilitator set grounds by asserting that participants were in the same room to learn from each other and that the wisdom was in the room. To enhance this, dialogue and participation in the workshop was guided by some key aspects including stepping up, stepping back; speaking from personal experiences; challenging ideas not people as well as considering and acknowledging impact as well as intent.

One of the things to ever remember is the distinction between teaching and facilitation. It was interesting to learn the job of the facilitator as being to create conducive environment for learning and guide participants to learn and develop desirable skills. This a meaningful leaning where learners participate in creating knowledge using their lived experiences and it matched well with what I learnt in early days when I joined teaching profession as our tutors told us that “you must be a guide at the side, not a sage on the stage.” Thus, introduction to facilitation skills activated my long stand understanding that learners are not “tabula-rasa” rather they have existing knowledge and experiences that can serve as a bridge to learning or acquisition of new knowledge. This corroborates the knowledge that facilitation is a step-wise process from “known to unknown” or “simple to complex.” I gained new insight during the session on application of the principles of backward design to create learner-centered content that experience alone is not enough rather it is important for learners to reflect on their experiences.

Another useful part of the facilitation training was the learning community which I regarded as co-facilitation for co-learning. Although this was not a new concept at all in my teaching experience for more than three decades, but it was an eye-opener on how learners can be effectively engaged in the form of “peer learning.” This is a very useful facilitation skill especially for public health postgraduate students who come for postgraduate education with vast experiences.

Knowledge and skills gained from the training are expected to be useful in the facilitation especially for undergraduate students. This is particularly in organizing group work for the students to reflect based on their experiences as well as using the role plays for the student to practice learned skills. Acquired knowledge and skills are most important when it comes to organizing and conducting continuing education and Professional development short courses and workshops in the University. Acquired knowledge will also be used in facilitating community rotation field works for undergraduate students where they work in groups for the sake of field experiential learning. In that context, the approach of the learning community gained from the training will be used to make students learn from each other, brainstorm ideas, share and exchange ideas, and appreciate different perspectives.

Emmanuel Balandya, MD PhD, Department of Physiology and Director of Postgraduate Studies, MUHAS, Tanzania.

Reflecting on my experiences during the Facilitation Workshop, a few things come to mind. First is the amount of effort that an effective facilitator must put in during preparation for the session, all in the best interest of the learners. It was clear that an effective facilitator goes over and above what is required. This requires an exceptional level of dedication to excellence.

Secondly, an effective facilitator does not go into a session with preconceived ideas regarding how the session should go, but rather with the goal to free learners’ minds and let their imaginations lead the way. To achieve this, an effective facilitator must be flexible to accommodate the unknowns.

To achieve learner-centeredness in the second point above, the facilitator must be effective at avoiding the center-stage. Various techniques can be utilized to achieve this, including assigning topics to learners in groups and letting them brainstorm, and thereafter discuss and present concepts. The facilitator may also use the technique of encouraging learners to dialogue among themselves rather than focus on the facilitator while presenting.

The fourth point pertains to the primary focus on the intended outcomes rather than processes. To achieve this, a facilitator begins by thinking about the outcomes of the session and walks backwards to devise methods and approaches, including innovations in case of challenges, to achieve the set outcomes through a process of “*backward design*.”

An effective facilitator must also be prepared to deliver content effectively amidst differences in approaches to learning by the different members of the group. To achieve this, the facilitator must therefore be aware that the learners may have different goals and expectations of the learning experience at baseline. Bringing these to attention at the beginning of a session may help in refining and harmonizing the goals across learners, hence opening up the minds of those who went in with minimal expectations. The facilitator must also have the skills to identify quick learners and distribute them across groups with the goal to bring other members of the group to speed through “*peer-to-peer*” learning. In the digital era, the facilitator must also be conversant with the use of online tools to manage multiple groups at once. And lastly, the facilitator must be patient enough but also familiar with techniques to handle difficult personalities in the group.

The skills that I have acquired during the Facilitation Workshop are invaluable and I am putting them into good use not only in managing educational seminars with students, but also in managing research groups.

Rifkatu Reng MBBS, FMCP, FACE, University of Abuja, Abuja, Nigeria.

My learning experience has been in gaining skills in mentoring and facilitation on discussion or training on a basis of co-learning disposition, thereby giving room for knowledge sharing across a divergent group of learners. This allows the flow of different learning experiences in the group to be tapped. The ability to be comfortable with silence as a facilitator to give a chance and sense of belonging for a co-mutual and psychosocial support for a mentee or learner to attain set out goals in academics and research by guidance was highly impactful.

As a facilitator I have gained understanding in ensuring that learning should be taken from the basics to the complex knowledge, built around the participants work or experience. A purposeful activity on mentorship in my department has been initiated, and mentees have been paired with available mentors.

## 7 Practical implications, objectives, and lessons learned

Capacity building in LMICs in biomedical research requires capacity building in associated professional skills like mentoring, communications, and leadership. Yet the majority of programs focus on building those skills without building the capacity to teach those skills within a local context and without external partnerships. Hence, an essential stage in building capacity is to develop a cohort of trained facilitators within the LMIC partner institutions who can contextualize and advance mentoring training within their cultural and institutional norms. To this end, we developed, delivered and assessed the outcomes of a 5-week course in the art and skill of facilitation to address a critical gap in capacity building in research mentor training. A central component of the course centered the explicit distinction between concepts of teaching and facilitation, using it to shift practice to learner-centered education. The course was further based on a modeling-practice-reflection pedagogical framework, and modeled inclusive facilitation practice, active engagement, learner agency development, and learning community and community of practice structures.

The concepts of power and positionality were used to take an equity and social justice perspective, and were themes that resonated with partners in Nigeria and Tanzania. Discussion of power and positionality developed around gender, age, and ethnic social identities and explored how those impact learning from both the facilitator and participant perspective. Discussion addressed multiple ways a facilitator can create effective learning when differentiated identities create a structural imbalance in the learning environment. These included multipartial facilitation, structured small group learning, and intentional discussion guidelines.

Small group facilitation engaged participants, expanded their knowledge and provided the opportunity for them to share their experiences and to think critically. Facilitation practices of learner-centered methods, giving everyone a voice/chance to talk, listening/being comfortable with silence and understanding the role of the facilitator were key takeaways for participants. Participants reported that they would soon apply these skills by facilitating open group discussions and giving learners the opportunity to express their ideas and share experiences, as well as facilitating small group work and using a backward design approach.

Our lessons learned are encouraged by the positivity of our initial, post-workshop evaluation, distal interviews, and autoethnographic descriptions that highlight key facilitation values, skills, and practices. Further, our delivery modality of a mix of asynchronous and online synchronous sessions was successful at developing perspectives and skills (Goldberg, B. B. et al., 2023). Based on the data from surveys, interviews and first-hand accounts, we are hopeful that the workshop will lead to institutionalization of effective professional skills facilitation as well as concomitant advances in learner-centered teaching practice.

Our model is replicable and adaptable to multiple LMIC settings. All that is required is a perspective shift from delivery of professional skills to capacity building in the training of professional skills and pedagogical expertise.

## 8 Conceptual and methodological constraints

The findings of this study need to be considered in light of several methodological limitations. We have a small, self-selected cohort of highly engaged participants from well-supported research capacity building programs in two large LMIC institutions in east and west Africa. Implementation of the program in a greater number of communities across a diversity of institutions would inform in a deeper way and provide valuable data on generalizability and scalability of the model. Our study also relies heavily on self-reported outcomes. A longitudinal study that observes participant skills directly and examines how participants implement mentor training within their local contexts and measures outcomes of those training sessions would provide more robust data on the efficacy of training and its longer-term impact. Such a study is currently in the planning phase.

## Supplementary Material

Supplementary material Table 2

Supplementary material Table 1

Supplementary material Training Slides

Supplementary Material Survey

## Data Availability

The raw data supporting the conclusions of this article will be made available by the authors, without undue reservation.

## References

[R1] Alidina S, Sydlowski MM, Ahearn O, Andualem BG, Barash D, Bari S, (2022). Implementing surgical mentorship in a resource-constrained context: a mixed methods assessment of the experiences of mentees, mentors, and leaders, and lessons learned. BMC Med. Educ 22:653. doi: 10.1186/s12909-022-03691-2 36045356 PMC9434847

[R2] Anderson LW, and Krathwohl D editors (2001). A Taxonomy for Learning, Teaching, and Assessing : a Revision of Bloom’s Taxonomy of Educational Objectives. New York, Longman.

[R3] Bonwell CC, and Eison JA (1991). Active learning: Creating excitement in the classroom. ASH#-ERIC Higher Education Report No. 1, Washington, D.C.: The George Washington University, School of Education and Human Development.

[R4] Brame C (2016). Active learning. Vanderbilt University Center for Teaching. Available at: https://cft.vanderbilt.edu/active-learning/ [Accessed 28, 2023].

[R5] Brookfield SD Becoming a critically reflective teacher. San Francisco: Jossey-Bass, (1995).

[R6] Brookfield SD (2003). “Understanding classroom dynamics: the critical incident questionnaire” in Teaching developmental writing: background readings. ed. Bernstein SN. 2nd Edn. (Boston, MA: Bedford/St., Martin’s Press)

[R7] Byars-Winston A, Rogers JG, Thayer-Hart N, Black S, Branshaw J, and Pfund C (2023). A randomized controlled trial of an intervention to increase cultural diversity awareness of research mentors of undergraduate students. Sci. Adv 9:eadf9705. doi: 10.1126/sciadv.adf9705 37224257 PMC10208566

[R8] Cole DC, Johnson N, Mejia R, McCullough H, Turcotte-Tremblay A-M, Barnoya J, (2016). Mentoring health researchers globally: diverse experiences, programmes, challenges and responses. Glob. Public Health 11, 1093–1108. doi: 10.1080/17441692.2015.1057091 26234691 PMC5020346

[R9] Cooke J (2005). A framework to evaluate research capacity building in health care. BMC Fam. Pract 6:44. doi: 10.1186/1471-2296-6-44 16253133 PMC1289281

[R10] Dancy M, Henderson C, Apkarian N, Johnson E, Stains M, Raker J, (2022). Physics instructors knowledge and use of active learning has increased over the last decade but most still lecture too much. arXiv: 2211.13082 [physics.ed-ph]

[R11] Deprez D, Busch AJ, Ramirez PA, Pedrozo Araque E, and Bidonde J (2023). Capacity-building and continuing professional development in healthcare and rehabilitation in low-and middle-income countries-a scoping review protocol. Syst. Rev 12:22. doi: 10.1186/s13643-023-02188-3 36814260 PMC9948347

[R12] Desai MM, Göç N, Chirwa T, Manderson L, Charalambous S, Curry LA, (2021). Strengthening the mentorship and leadership capacity of HIV/AIDS and tuberculosis researchers in South Africa. Am. J. Trop. Med. Hyg 105, 1317–1325. doi: 10.4269/ajtmh.21-0072 34398822 PMC8592193

[R13] Dewey J (1938). Experience and education. Educ. Forum 50, 241–252. doi: 10.1080/00131728609335764

[R14] Ellis C, Adams T, and Bochner AP (2010). Autoethnography: an overview. Forum qualitative Sozialforschung/forum: Forum Qual. Soc. Res, 12:10. doi: 10.17169/fqs-12.1.1589

[R15] Franzen SRP, and Chandler C (2017). Lang THealth research capacity development in low and middle income countries: reality or rhetoric? A systematic meta-narrative review of the qualitative literature. BMJ Open 7:e012332. doi: 10.1136/bmjopen-2016-012332 PMC5278257 28131997

[R16] Freeman S, Eddy SL, McDonough M, Smith MK, Okoroafor N, Jordt H, (2014). Active learning increases student performance in science, engineering, and mathematics. Proc. Nat. Acad. Sci. U. S. A 111, 8410–8415. doi: 10.1073/pnas.1319030111 PMC4060654 24821756

[R17] Freire P, and Ramos MB (1970). Pedagogy of the oppressed. New York, Seabury Press.

[R18] Gandhi M, Raj T, Fernandez R, Rispel L, Nxumalo N, Lescano AG, (2019). Mentoring the mentors: implementation and evaluation of four Fogarty-sponsored mentoring training workshops in low-and middle-income countries. Am. J. Trop. Med. Hyg 100, 20–28. doi: 10.4269/ajtmh.18-0559 30430977 PMC6329359

[R19] Garba SJ (2012). The impact of colonialism on Nigerian education and the need for E-learning technique for sustainable development.

[R20] Giacomini NG, and Schrage JM (2009). Reframing campus conflict: student conduct practice through a social justice lens. 1st Edn. Sterling, VA: Stylus Publishing.

[R21] Goldberg BB, Bruff DO, Greenler RM, Barnicle K, Green NH, Campbell LE, (2023). Preparing future STEM faculty through flexible teaching professional development. Plos one. 18, e0276349. 37824586 10.1371/journal.pone.0276349 PMC10569627

[R22] Hamer DH, Hansoti B, Prabhakaran D, Huffman MD, Nxumalo N, Fox MP, (2019). Global Health research mentoring competencies for individuals and institutions in low-and middle-income countries. Am. J. Trop. Med. Hyg 100, 15–19. doi: 10.4269/ajtmh.18-0558 30430976 PMC6329357

[R23] Handelsman J, Elgin S, Estrada M, Hays S, Johnson T, Miller S, (2022). Achieving STEM diversity: Fix the classrooms. Science. 376, 1057–1059. doi: 10.1126/science.abn9515 35653460

[R24] Hansoti B, Kalbarczyk A, Hosseinipour MC, Prabhakaran D, Tucker JD, Nachega J, (2019). Global Health mentoring toolkits: a scoping review relevant for low-and middle-income country institutions. Am. J. Trop. Med. Hyg 100, 48–53. doi: 10.4269/ajtmh.18-0563 30430981 PMC6329353

[R25] Hargreaves S, Rustage K, Nellums LB, Bardfield JE, Agins B, Barker P, (2019). Do quality improvement initiatives improve outcomes for patients in antiretroviral programs in low-and middle-income countries? A systematic review. J. Acquir. Immune Defic. Syndr 81, 487–496. doi: 10.1097/QAI.0000000000002085 31149954 PMC6738622

[R26] Health research mentorship in low and middle-income countries (HERMES) (2022). A TDR global practical guide to spur mentorship institutionalization. Geneva: World Health Organization; License: CC BY-NC-SA 3.0 IGO. Available at: https://apps.who.int/iris/bitstream/handle/10665/363381/9789240058675-eng.pdf.. (Accessed Feburary 1, 2023).

[R27] Heimburger DC, Riley LW, Thomas Y, Cohen CR, Vermund SH, Bale K, (2016). The National Institutes of Health Fogarty International Center Global Health scholars and fellows program: collaborating across five consortia to strengthen research training. Am. J. Trop. Med. Hyg 95, 728–734. doi: 10.4269/ajtmh.16-0190 27382074 PMC5014285

[R28] Hokanson SC, and Goldberg BB (2018). “Proactive postdoc mentoring” in Book chapter in the postdoc landscape: The invisible scholars. eds. Jaeger AJ and Dinin AJ (Cambridge, Mass: Academic Press). 91–120. doi: 10.1016/B978-0-12-813169-5.00005-7

[R29] Kay S, and Nystrom B (1971). Education and colonialism in Africa: an annotated bibliography. Comp. Educ. Rev 15, 240–259. doi: 10.1086/445535

[R30] Kirkpatrick DL. Evaluating training programs: The four levels. San Francisco, CA: Berrette-Koehler; (1994).

[R31] Kirkpatrick D (1996). Great ideas revisited. Techniques for evaluating training programs. Revisiting Kirkpatrick’s four-level model. Train Dev 50(1): 54–59. Available at: https://eric.ed.gov/?id=EJ515660 [Accessed January 17, 2022].

[R32] Lescano AG, Cohen CR, Raj T, Rispel L, Garcia PJ, Zunt JR, (2019). Strengthening mentoring in low-and middle-income countries to advance Global Health research: an overview. Am. J. Trop. Med. Hyg 100, 3–8. doi: 10.4269/ajtmh.18-0556 PMC6329352 30430982

[R33] Light G, Cox R, and Calkins SC (2009). Learning and teaching in higher education: The reflective professional. 2nd ed Edn. Thousand Oaks, CA: Sage Publishing.

[R34] National Academies of Sciences, Engineering, and Medicine. (2019). The science of effective mentorship in STEMM. Washington, DC: The National Academies Press. 31958221

[R35] Noormahomed E, Williams P, Lescano AG, Raj T, Bukusi EA, Schooley RT, (2019). The evolution of mentorship capacity development in low-and middle-income countries: case studies from Peru, Kenya, India, and Mozambique. Am. J. Trop. Med. Hyg 100, 29–35. doi: 10.4269/ajtmh.18-0560 30430979 PMC6329354

[R36] Oppong E, Bao H, Tang W, Echavarria Mejia MI, Glozah F, Asanga N, (2021). A global crowdsourcing open call to improve research mentorship in low-and middle-income countries: a mixed methods analysis. Am. J. Trop. Med. Hyg 106, 250–256. doi: 10.4269/ajtmh.21-0607 34662869 PMC8733547

[R37] Pfund C, Byars-Winston A, Branchaw J, Hurtado S, and Eagan K (2016). Defining attributes and metrics of effective research mentoring relationships. AIDS Behav 20, 238–248. doi: 10.1007/s10461-016-1384-z 27062425 PMC4995122

[R38] Pfund C, House SC, Asquith P, Fleming MF, Buhr KA, Burnham EL, (2014). Training mentors of clinical and translational research scholars: a randomized controlled trial. Acad. Med 89, 774–782. doi: 10.1097/ACM.0000000000000218 24667509 PMC4121731

[R39] Pfund C, Spencer KC, Asquith P, House SC, Miller S, and Sorkness CA (2017). Building national capacity for research mentor training: an evidence-based approach to training the trainers. CBE—Life Sci. Educ 14:ar24. doi: 10.1187/cbe.14-10-0184 PMC4477740 26033872

[R40] Potter C, and Brough R (2004). Systemic capacity building: a hierarchy of needs. Health Policy Plan. 19, 336–345. doi: 10.1093/heapol/czh038 15310668

[R41] Program Evaluation Core (2023). Northwestern University. Available at: https://www.northwestern.edu/programevaluationcore/

[R42] Rogers J, Sorkness C, Spencer K, and Pfund C (2018). Increasing research mentor training among biomedical researchers at clinical and translational science award hubs: the impact of the facilitator training initiative. J. Clin. Transl. Sci 2, 118–123. doi: 10.1017/cts.2018.33 30370062 PMC6199543

[R43] Rose E, Gavarkavich Diane, Nzala Selestine H., (2022). Elevating mentorship competency for sustained impact through the University of Zambia Mentor Training Program, 05 may 2022, PREPRINT (version 1) available at Research Square. 10.4269/ajtmh.22-0726 PMC10397461 37400065

[R44] Rose ES, Nzala SH, Goma FM, Gavarkavich D, Deepak A, Parker OJ, (2023). Elevating Mentorship Competency for Sustained Impact via the University of Zambia Mentor Training Program. Am J Trop Med Hyg 109, 489–494. doi: 10.4269/ajtmh.22-0726 37400065 PMC10397461

[R45] Routenberg R, Thompson E, and Waterberg R (2013). “When neutrality is not enough: wrestling with the challenges of multipartiality” in The art of effective facilitation: Reflections from social justice educators ed. Landreman LM (Sterling, VA: Stylus Publishing), 116–129.

[R46] Spencer KC, Melissa Mcdaniels, Utzerath, Emily, Rogers, Jenna Griebel, Sorkness Christine A., Asquith Pamela, and Pfund Christine (2018). Building a sustainable National Infrastructure to expand research Mentor training, CBE—Life sciences education Vol 17, No. 3. 10.1187/cbe.18-03-0034 PMC6234808 30153422

[R47] Sun T, Drane D, McGee R, Campa III H, Goldberg BB, Hokanson SC, (2023). A national professional development program fills mentoring gaps for postdoctoral researchers. Plos one 18:e0275767. 37315043 10.1371/journal.pone.0275767 PMC10266628

[R48] Wandela Lucas, and Eugenia, (2014). “Tanzania post-colonial educational system and perspectives on secondary science education, pedagogy, and curriculum: A qualitative study” College of Education Theses and Dissertations 71. DePaul University.

[R49] Wiggins GP, and McTighe J (2005). Understanding by design (2nd ed.)Pearson.

[R50] Womack VY, Wood CV, House SC, Quinn SC, Thomas SB, McGee R, (2020). Culturally aware mentorship: lasting impacts of a novel intervention on academic administrators and faculty. PLoS One 15:e0236983. doi: 10.1371/journal.pone.0236983 32764768 PMC7413486

[R51] Zappella N (2007). Balancing social power in dialogue: what it means to be a multipartial facilitator in intergroup dialogues. Ann. Arbor

[R52] Zunt JR, Chi BH, Heimburger DC, Cohen CR, Strathdee S, Hobbs N, (2016). The National Institutes of Health Fogarty International Center Global Health Scholars and Fellows Program: Collaborating Across Five Consortia to Strengthen Research Training. Am J Trop Med Hyg 95, 728–734. 27382074 10.4269/ajtmh.16-0190 PMC5014285

