# Supplementary material for: Training in the art and science of facilitation to scale research mentor training in low and middle income countries
Source: Front Educ (Lausanne). Author manuscript; Available in PMC 2024 Jun 6. (PMC11155035; doi:10.3389/feduc.2023.1270480)
Supplement: Supplementary material Table 2 [file NIHMS1994830-supplement-Supplementary_material_Table_2.docx]

**Run-of-show:**

| **Facilitator** | **Timing** | **Slides** | **Activity/topic** |
| --- | --- | --- | --- |
|  | 0-5 |  | Folks arrive. Set up and Introductions, Goals of the session; session norms; Overall framing of the project; |
|  | 5-10 |  | Prompt; initial work; recap or new material overview |
|  | 10-25 |  | Activity 1: Breakout at tables; group report and synthesis |
|  | 25-35 |  | Setup second major learning goal; Detailed look at our evaluation and cycles of improvement; Prompt |
|  | 35-45 |  | Activity 2: In pairs, turn to your neighbor for think-pair-share: Share a one-pager of the example of feedback from… |
|  | 45-55 |  | Questions and next steps |
| Total | 55 |  |  |
