# Supplementary material for: Training in the art and science of facilitation to scale research mentor training in low and middle income countries
Source: Front Educ (Lausanne). Author manuscript; Available in PMC 2024 Jun 6. (PMC11155035; doi:10.3389/feduc.2023.1270480)
Supplement: Supplementary material Table 1 [file NIHMS1994830-supplement-Supplementary_material_Table_1.docx]

**Supplemental Information**

Facilitation Training Post-Workshop Interview Protocol

Introduction:

You have been asked to take part in this interview since you completed the facilitation training workshop series with Dr. Bennett Goldberg. The purpose of this follow-up interview is to gain further insight into the effectiveness of the workshop for each participant. You will also be asked to comment on any parts of the workshop that could be improved.

There are eight questions in this interview. If you do not feel comfortable answering a question, you may decline to answer it. At the conclusion of all interviews, the results will be combined in a summary report. Please be as honest and thoughtful about your responses as possible. Your feedback is important because it will help Dr. Goldberg improve his facilitation practices for future workshops.

Interview questions:

1. Reflecting back on how you felt before the training, how effective was the training in building your confidence in and awareness of facilitation practices?
2. Teaching means different things to different people, what does it mean to you?
   1. What are the commonalities and differences between teaching and facilitation?
   2. What are the commonalities and differences between facilitation and learning?
3. Which of your facilitation skills were best developed during the training?
4. The facilitation approach that you learned is developed in the space of Western higher education, but it is used in many places throughout the world. How do you think the students who you teach in Nigeria/Tanzania will respond to this approach?
5. Where, in your context, are you planning to use facilitation skills?
6. The training combined faculty from Tanzania and Nigeria. What was it like for you to work with faculty from Tanzania? (or Nigeria)?
   1. Did doing the training with faculty from Tanzania/Nigeria add any value to the training? Please explain.
7. In your opinion, what was the most valuable part of this training?
8. Please share any suggestions you have for how the training could be improved.
