# Supplementary material for: Training in the art and science of facilitation to scale research mentor training in low and middle income countries
Source: Front Educ (Lausanne). Author manuscript; Available in PMC 2024 Jun 6. (PMC11155035; doi:10.3389/feduc.2023.1270480)

TZ/NG Facilitator Training

Bennett Goldberg, PhD
Professor of Physics and Astronomy
Faculty Director, Program Evaluation Core
Former Director, Searle Center for Advancing Learning and Teaching

Day 1/4, August 10, 2022

Northwestern University

University of Abuja

Dr. Fatima Kyari & Dr. Rifkatu Mshelia-Reng

Muhimbili University of Health and Allied Sciences

Dr. Emmanuel Balandya & Dr. Deodatus Kakoko

University of Ilorin

University of Ibadan

Catholic University of Health and Allied Sciences

We Learn Together

We are here together in the same rooms to learn from each other. The wisdom is in the room.

We seek to explore new ideas, practice together, role play, in the process of developing facilitation skills.

We are aware of and acknowledge the power and positionality differential, and seek that such differential is, when necessary, overcome to enhance learning.

Framing

- Who we are
- What we seek to achieve
- How we will achieve it
- Google and mechanics

Who we are

- Faculty from ….MUHAS, Abuja, Ilorin, Ibadan & CUHAS
- Brief Introductions: Name, institution, the workshop, teaching or training where you plan to facilitate

Our Goal…

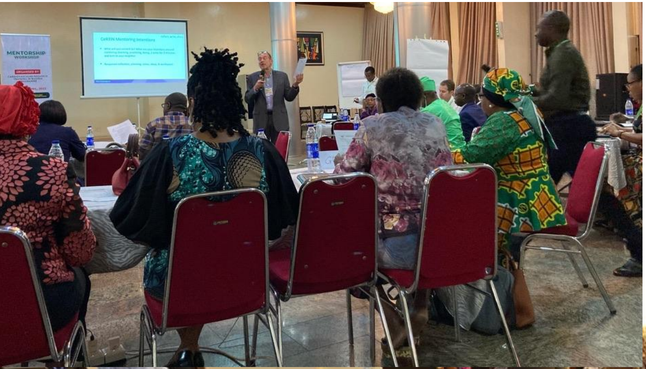

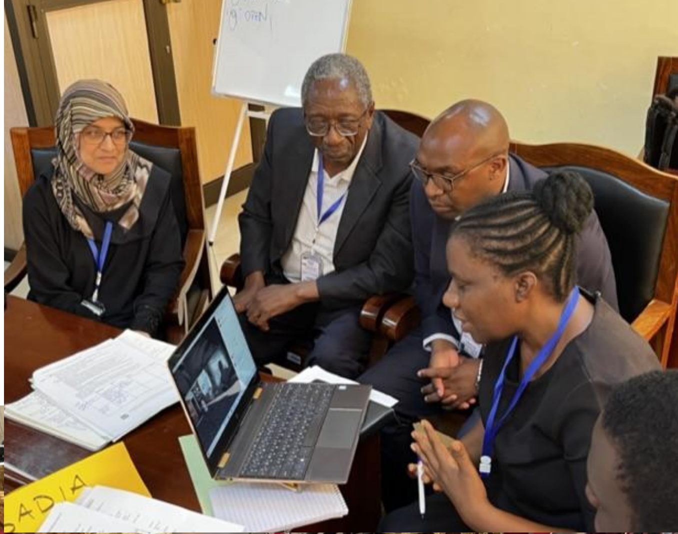

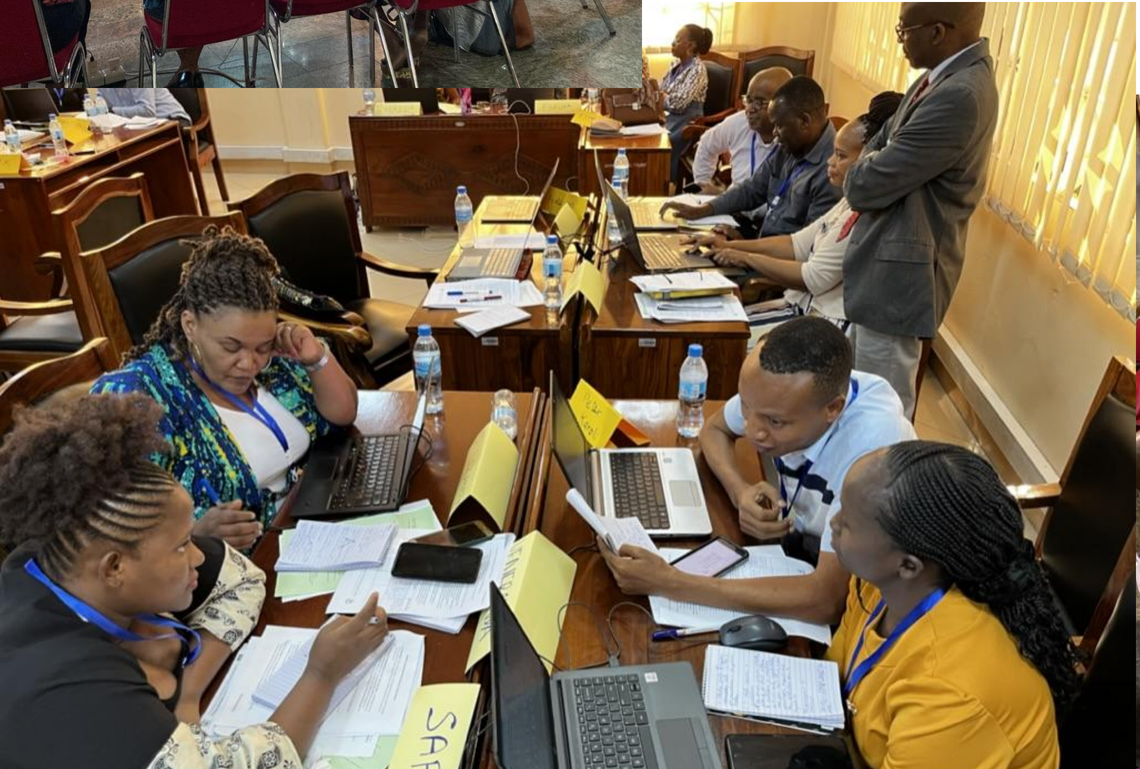

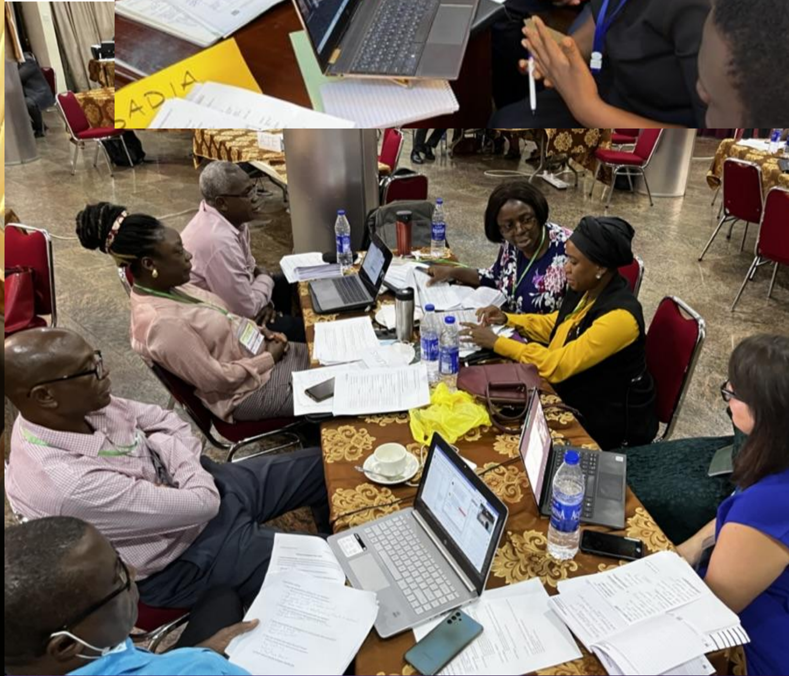

Breakout 1:
Your expertise, experiences, and assets

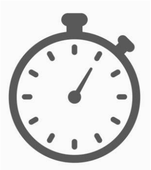

8 min

- Introductions to each other
- Name & Work context – department /program
- What expertise do you bring, *as a facilitator*, and what are you looking forward to learning?

Your expectations are....

expand and strengthen my facilitation skills.

to have a structure and know the technicalities in planning, meetings and post-meeting assessment and feedback.

apply lessons and skills learnt in my role as a teacher, a mentor, and an advocate for better healthcare (education, service delivery, financing, etc). These will be while teaching, training or even chairing educational/project and board meetings; and also at home!

improve participation from everyone in the room, including the quieter voices and/or marginalised people; especially in considering hierarchical roles that is prevalent in our culture and in the medical profession

I expect to learn additional skills for facilitation of large and small teaching classes

Learning Objectives

Participants in this short course will…

1. be able to describe the foundational elements of effective facilitation, the key features of active learning and high engagement, and apply the principles of backward design to create learner-centered content and experiences.
2. be able to develop and produce a small group learning interaction; be able to describe the key steps and processes, identify facilitation challenges and opportunities, and support multiple interactions.
3. be able to identify appropriate areas of assessment and evaluation of effective facilitation and use these in an observation rubric; be able to produce and perform one's own designed small group learning interaction; be able to learn from peer- and expert evaluation.

Methods

Modeling

- Demonstration
- Observation
- Experience
- Practical

Reflection

- Understanding
- Frameworks
- Context
- Generalize

Practice

- Application
- Repetition
- Assessment

Guidelines for dialogue and participation

- Step up, step back
- Speak from your own experiences
- Challenge ideas, not people
- Consider and acknowledge impact as well as intent

Mute microphone if not speaking

Feel free to turn on video

Use the chat for questions or comments

“Raise hand” if you want to unmute

Google and mechanics

- Shared Google folder link here
  - Bookmark it; also in every session invite
  - Note everyone has editor access - so feel free to write, but do not delete…
  - Make copies, download for your interest and future use

Breakouts aka small groups

- Be present (as much as you can)
- Participate in discussion (take turns)
- Observational and active listening is…
- Notes and ideas in common google-docs
- Random for sessions 1 & 2, Fatima, Emmanuel, Rifkatu & Deodatus in rooms to help
- With colleagues in sessions 3 & 4

Breakout 2: What is facilitation?

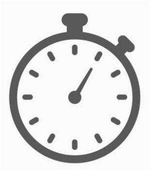
8 min

How is facilitation different than teaching?

How is facilitation like teaching?

[Notes document]

Facilitation

Facilitation is the act of helping other people to deal with a process or reach an agreement or solution without getting directly involved in the process, discussion, etc. yourself.

Faculty Approaches to Teaching

The Transmission Approach

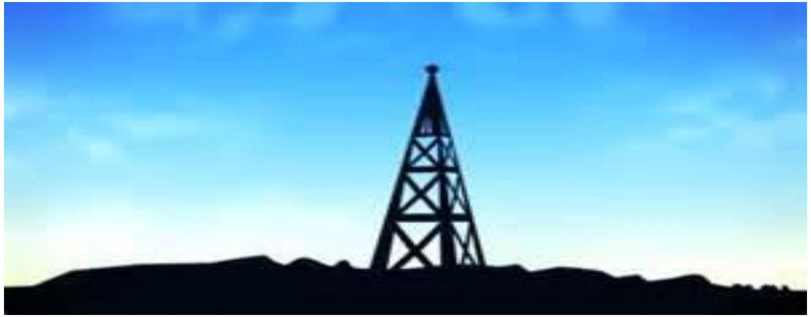

Teaching Intention:

- To transfer or transmit the course content
- To get it *out* (& hopefully in) to students

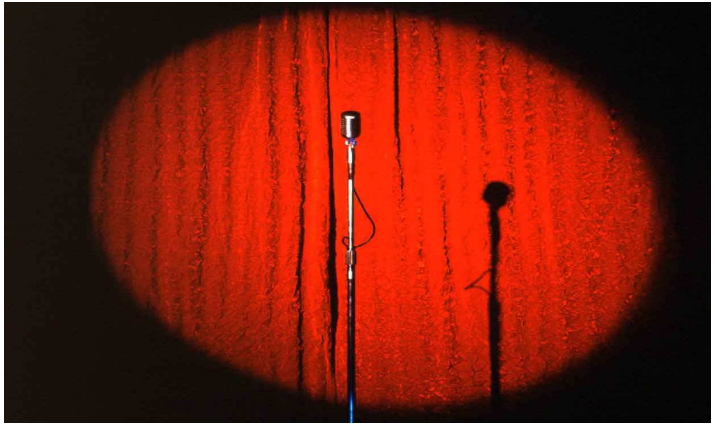

Implicit Assumption

- Teaching as monologue
- One dimensional, limited

Relationship to learning:

Teaching is *separate* from learning

(Light G & Cox R, Calkins, S. (2009); Prosser M. & Trigwell K 1999)

Faculty Approaches to Teaching

The Acquisition Approach

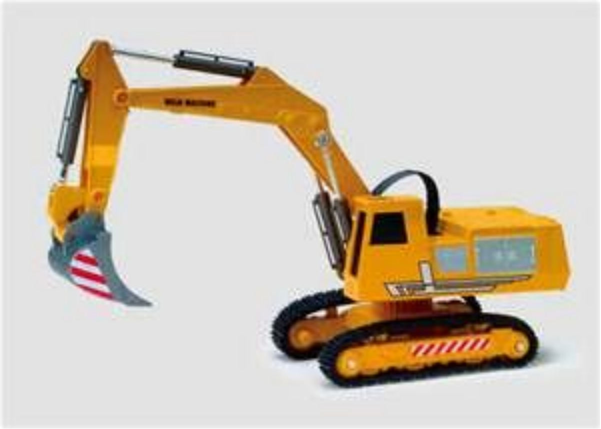

Relationship to learning

Teaching causes learning

Teaching Intention

- Get course content out & ‘in’ to students
- Help students acquire content of course (tools, knowledge, skills)

Implicit Assumption

- Teaching as instruction (or explanation)
- One-way, linear

(Light G & Cox R, Calkins, S. (2009); Prosser M. & Trigwell K 1999)

Faculty Approaches to Teaching

The Engaged Approach

Teaching Intention

- Share course content with students
- Get students to reconstruct knowledge for themselves

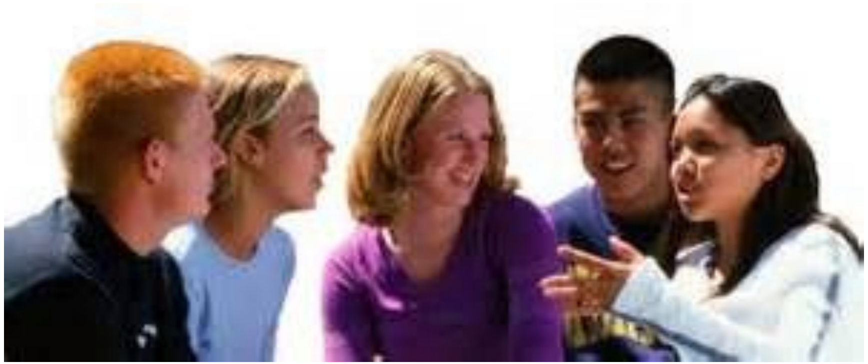

Implicit Assumption

- Teaching as dialogue
- 2-way conceptual exchange & change

Relation to Learning

Teaching is *by-product* of learning

(Light G & Cox R, Calkins, S. (2009); Prosser M. & Trigwell K 1999)

Faculty Approaches to Teaching

	**Transmission**	**Acquisition**	**Engaged**
**Focus**	Content-***teacher***	Content-***student***	Content-***learning***
**Teaching intention**	•To transfer or transmit the course content<br>•To get it *out* (& hopefully in) to students	•Get course content out & 'in' to students<br>•Help students acquire content of course (tools, knowledge, skills)	•Share course content with students<br>•Get students to reconstruct knowledge for themselves
**Implicit assumption**	•Teaching as monologue<br>•One dimensional, limited	•Teaching as instruction (or explanation<br>•One-way, linear	•Teaching as dialogue<br>• 2-way conceptual exchange & change
**Relationship to learning**	Teaching is *separate* from learning	Teaching *causes* learning	Teaching is *by-product* of learning

(Light G & Cox R, Calkins, S. 2009; Prosser M. & Trigwell K 1999)

So what do we mean by learning?

↓

Student & Adult Approaches to & Conceptions of Learning

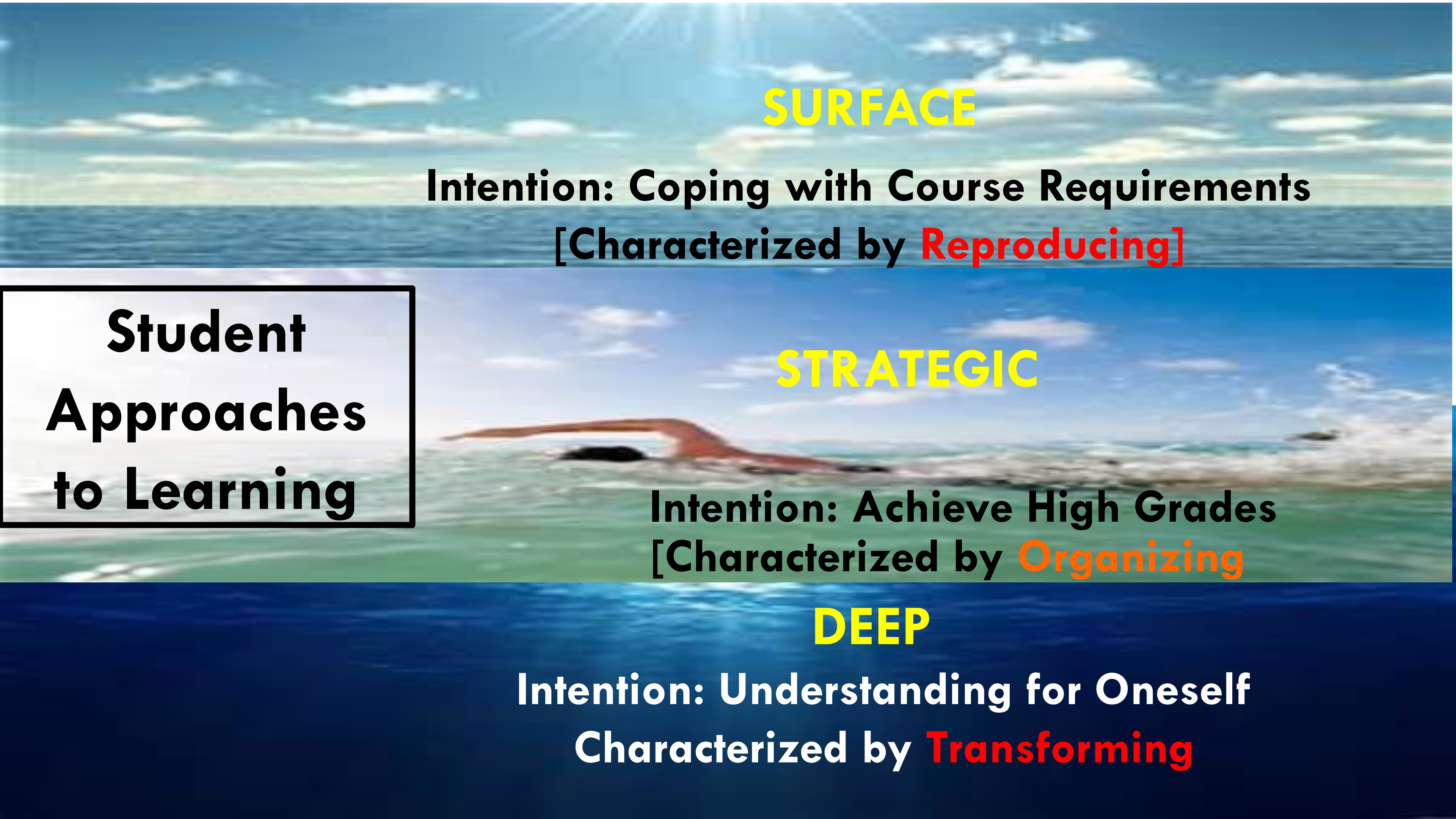

Student Approaches to Learning
SURFACE
Intention: Coping with Course Requirements
[Characterized by Reproducing]
STRATEGIC
Intention: Achieve High Grades
[Characterized by Organizing
DEEP
Intention: Understanding for Oneself
Characterized by Transforming

What else do we know about deep learning?

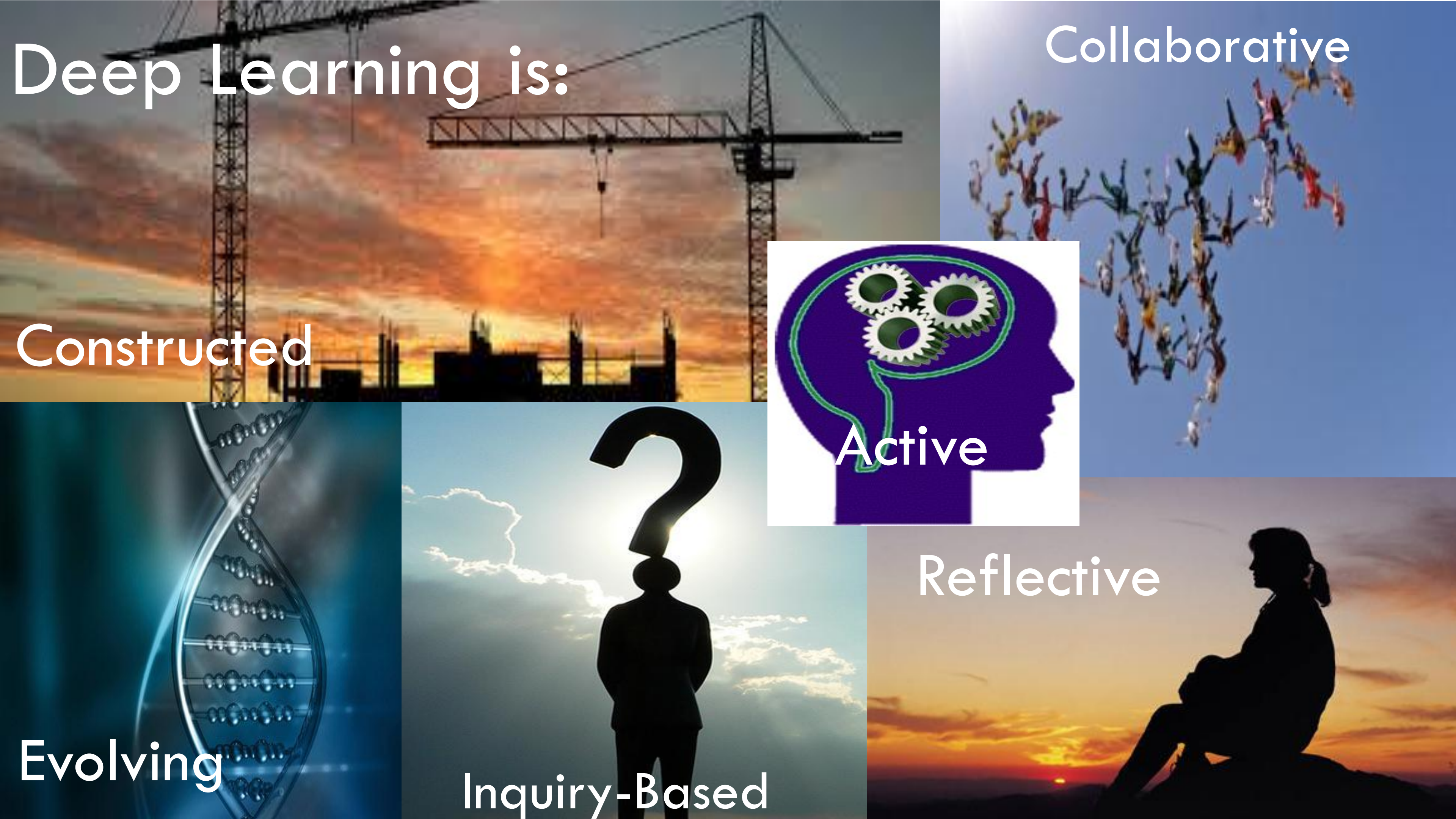
Deep Learning is:
Collaborative
Constructed
Active
Reflective
Evolving
Inquiry-Based

The Role of Reflection in Learning

"We do not learn from experience…we learn from reflecting on experience."

-John Dewey
Social Constructivist Theory of Learning

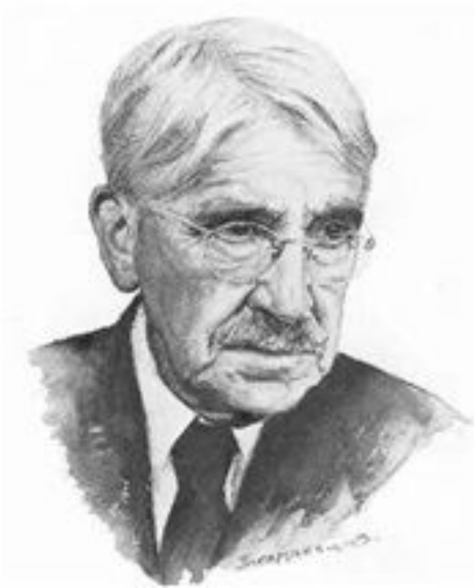

What kind of thinking do you want from your participants?

Bloom's Revised Taxonomy (Cognitive)

Creating Generating new ideas, products, or ways of viewing things
Designing, constructing, planning, producing, inventing

Evaluating Judging based on criteria
Experimenting, checking, hypothesizing, critiquing, justifying

Analyzing Breaking information into parts to explore relationships
Comparing, organizing, deconstructing, interrogating, finding

Applying Using information in another familiar situation
Implementing, carrying out, using, executing

Understanding Explaining ideas or concepts
Interpreting, summarizing, paraphrasing, classifying, explaining

Remembering Recalling information
Recognising, listing, describing, retrieving, naming, finding

Anderson & Krathwohl, 2001

Breakout 3

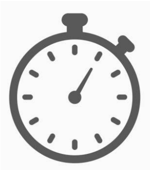
10 min

As a facilitator…

What do you do when someone asks you a question?

[Notes here]

For next time…

- Short reading, short video or two and reflection
- Look for email on Friday, reminder on Monday

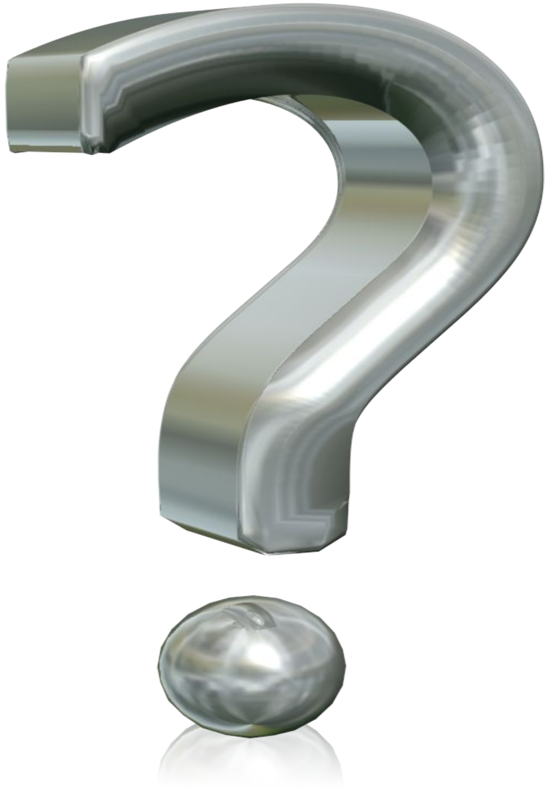

Fundamentals of Inclusive Facilitation

- Reflect on yourself
  - What power and positionality do you have in the room? What might be the impacts?
- Reflect on your participants
  - What power and positionality do your participants have in the room? How might that impact the learning process?
- Practice multipartiality
  - What are the dominant narratives and what techniques can you apply?

Power and positionality

Power Defined

Power is diffuse, dynamic, and relational. It can facilitate or restrict the ability of an individual to influence or act within a classroom or research context.

"All pedagogies... enact understandings about how knowledge is constructed, what counts as valid knowledge, and who can create or contribute to knowledge."

(Lee *et al.*, 2012)

Positionality defined

One’s social location or position assigned and negotiated as a result of combining various social factors or identifiers; including but not limited to:

> race, sex, class, gender, ability, age, religion, sexual orientation, nationality, physical stature, education, occupation, relational status, language.

Hearn, “Positionality, Intersectionality and Power,” 42

Breakout 4:
Power & Positionality in Mentoring

12 min

- Spend 5 minutes writing on your paper how issues of power and positionality may impact your facilitation from your perspective
- Consider issues of status, age, gender, and others that are important in your context
- Share with a neighbor

Roles of Facilitators

Make it safe: Take time to tell/show the group members that the workshop is a safe place to be honest about their ideas and feelings. Everyone's ideas are worth hearing.

Keep it constructive and positive: Remind members of your group to keep things positive and constructive. Ask the group how they want to deal with negativity and pointless venting. Remind them that the seminar is about working together to learn, not complaining about their current situation or discounting the ideas of others in the interest of a personal agenda.

Make the discussion functional: At the start of each session, explain the goals of the session to the group. Try to keep the group on task without rushing them. If the conversation begins to move beyond the main topic, bring the discussion back to the main theme of the session.

Give members of the group functional roles and responsibilities: Assign or ask for volunteers to take notes, keep track of time, and report out in the larger group at the end of the session. Functional roles help keep participants engaged.

Give all participants a voice: In a group, there are likely to be issues of intimidation and power dynamics that can play out in ways that allow certain members of the group to dominate and others to remain silent. At the start of the conversation, mention that the group is mixed by design, and point out that a diversity of perspectives is an essential part of the process. Remind group members to respect all levels of experience. It's important that everyone's voice is heard!

NG/TZ Facilitator Training

Bennett Goldberg, PhD
Professor of Physics and Astronomy
Faculty Director, Program Evaluation Core
Former Director, Searle Center for Advancing Learning and Teaching

Day 1/2, August 16 or 19, 2022

Northwestern University

University of Abuja

Dr. Fatima Kyari & Dr. Rifkatu Mshelia-Reng

Muhimbili University of Health and Allied Sciences

Dr. Emmanuel Balandya & Dr. Deodatus Kakoko

University of Ilorin

University of Ibadan

Catholic University of Health and Allied Sciences

We Learn Together

We are here together in the same rooms to learn from each other. The wisdom is in the room.

We seek to explore new ideas, practice together, role play, in the process of developing facilitation skills.

We are aware of and acknowledge the power and positionality differential, and seek that such differential is, when necessary, overcome to enhance learning.

Guidelines for dialogue and participation

- Step up, step back
- Speak from your own experiences
- Challenge ideas, not people
- Consider and acknowledge impact as well as intent

Mute microphone if not speaking

Feel free to turn on video

Use the chat for questions or comments

“Raise hand” if you want to unmute

Methods

Modeling

- Demonstration
- Observation
- Experience
- Practical

Reflection

- Understanding
- Frameworks
- Context
- Generalize

Practice

- Application
- Repetition
- Assessment

Breakout 3

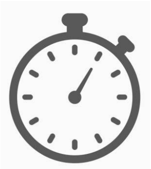

10 min

As a facilitator…

What do you do when someone asks you a question?

Explore all options. Describe the purpose of your various options.

[Notes here]

Reflect on part 1 of pre-work

- What is active learning?
- What are the steps, generally, of peer-instruction as demonstrated in the videos?

Key aspects of active learning...

Active learning helps students:

- Think for themselves
- Make connections
- Raise questions
- Reflect
- Collaborate with peers
- Communicate
- Solve problems
- Make decisions

So the instructor needs to ...

- Frame authentic questions
- Allow students time and space to think and share
- Create activities and assignments that promote meaningful learning
- Construct pairs, groups and teams

Active Learning Strategies…a Continuum

Simple Tasks
(short & relatively unstructured)

↔

Complex Tasks
(Longer duration; more carefully structured)

One strategy is not inherently “better” than another; it just depends on your learning goals for the course or session

Bonwell, Charles C.; Eison, James A. Active Learning: Creating Excitement in the Classroom. 1991 ASHE-ERIC Higher Education Reports.

Active Learning Strategies

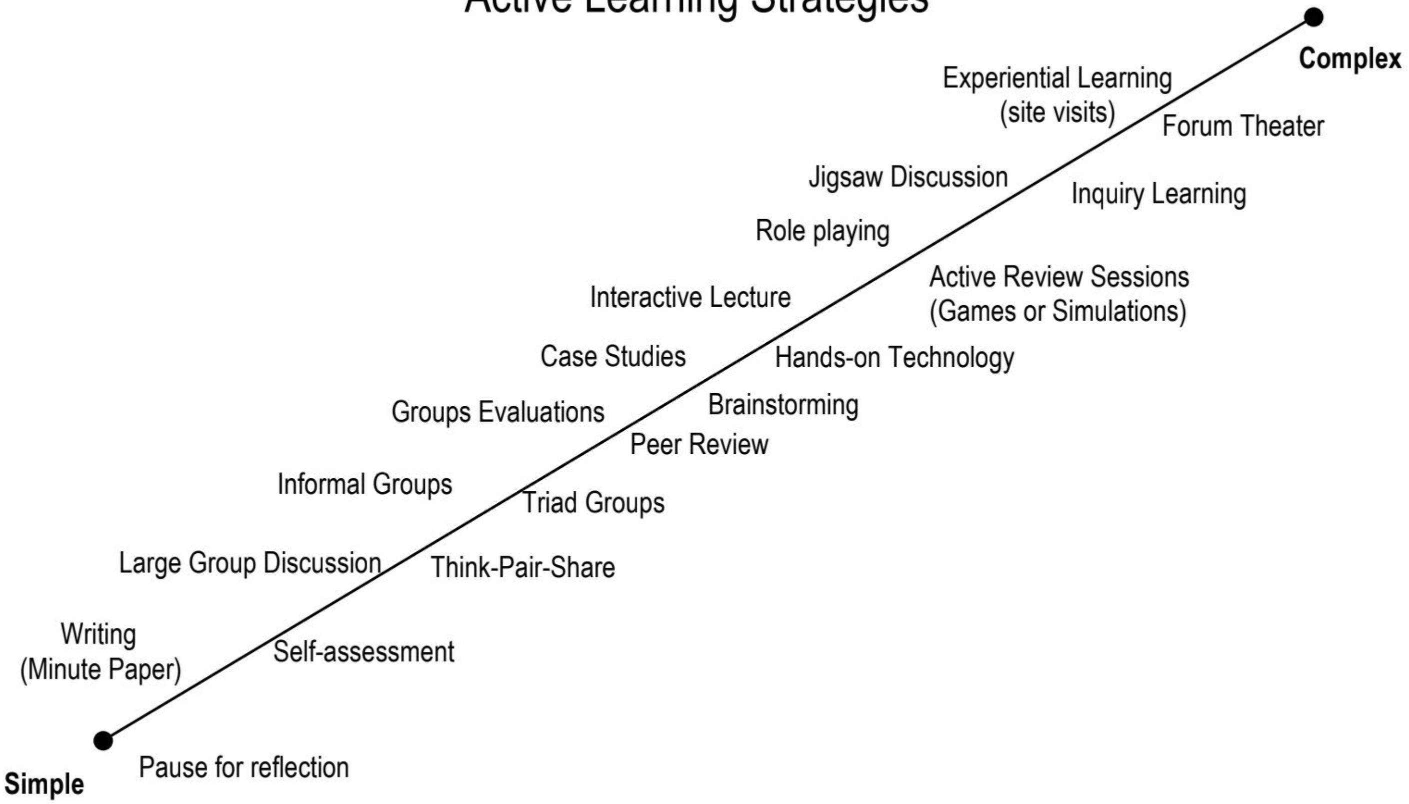

This is a spectrum of some active learning activities arranged by complexity and classroom time commitment.

Prepared by Chris O'Neal and Tershia Pinder-Grover, Center for Research on Learning and Teaching, University of Michigan

Backward Faded Scaffolding

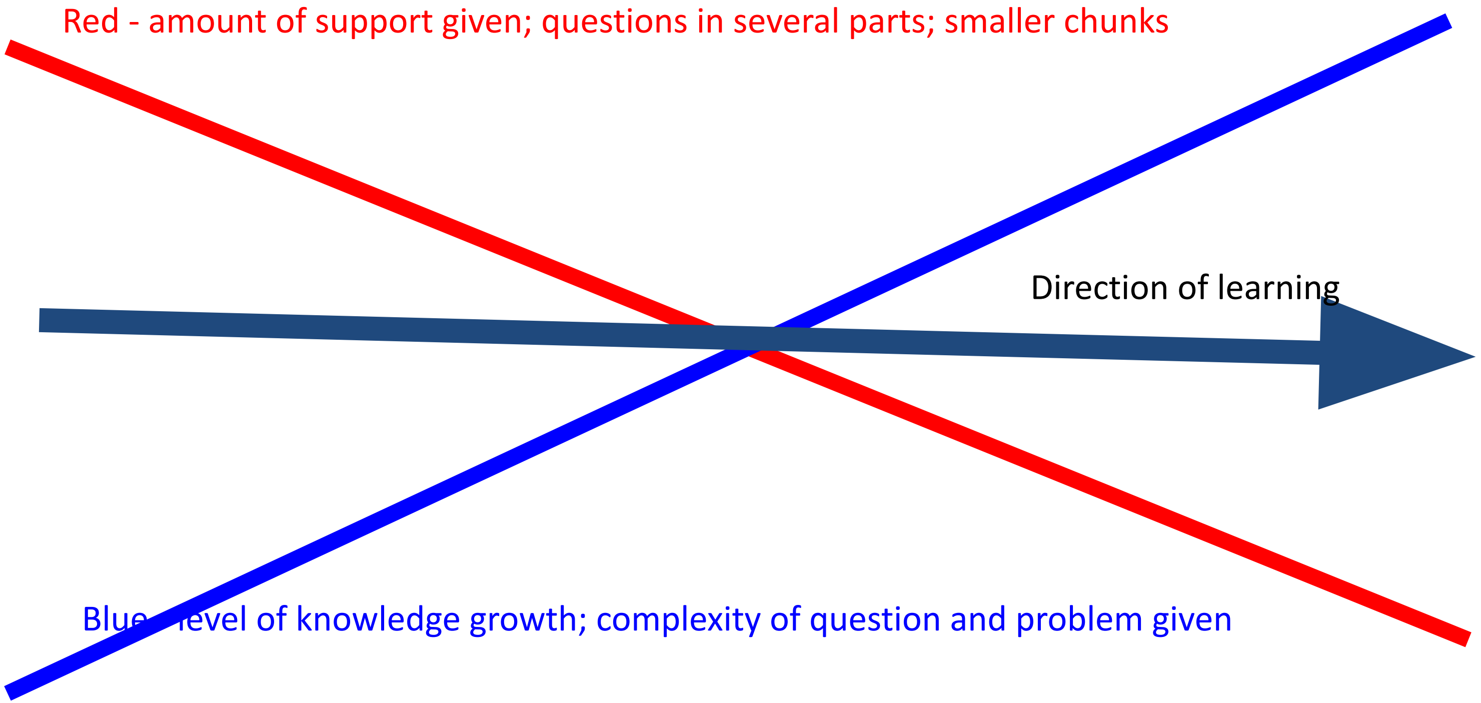

Fundamentals of Inclusive Facilitation

- Reflect on yourself
  - What power and positionality do you have in the room? What might be the impacts?
- Reflect on your participants
  - What power and positionality do your participants have in the room? How might that impact the learning process?
- Practice multipartiality
  - What are the dominant narratives and what techniques can you apply?

Establishing Brave Spaces: The roles of safety and comfort in dialogue

Adapted from the Program on Intergroup Relations

University of Michigan 2008

Aro, B. & Clemens, K. From Safe Spaces to Brave Spaces: A new way to frame dialogue around diversity and social justice *The Art of Effective Facilitation*

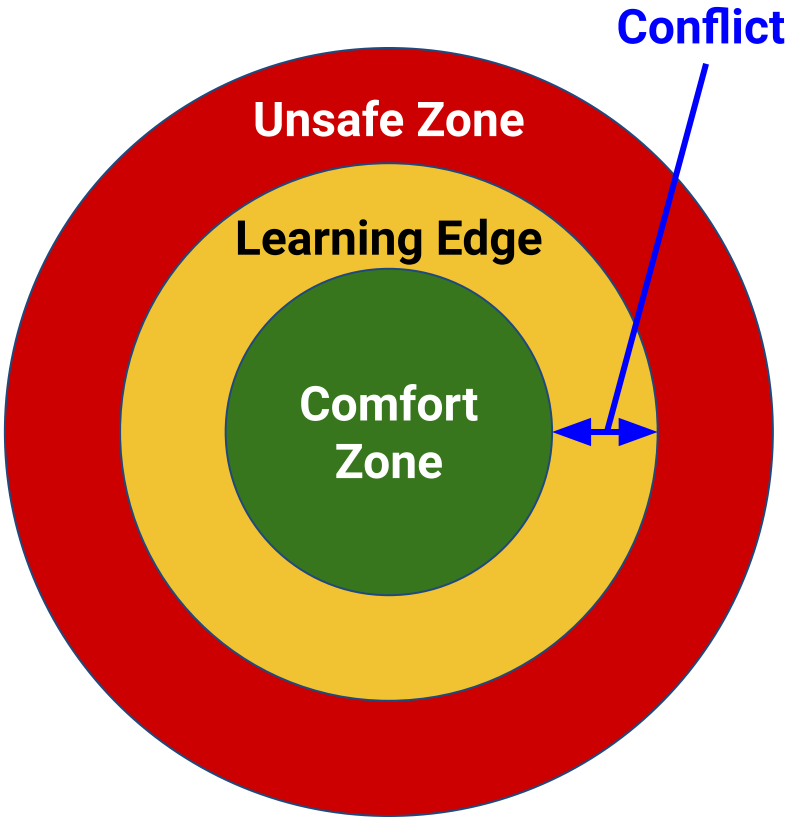

Power and positionality

Power Defined

Power is diffuse, dynamic, and relational. It can facilitate or restrict the ability of an individual to influence or act within a classroom or research context.

“All pedagogies... enact understandings about how knowledge is constructed, what counts as valid knowledge, and who can create or contribute to knowledge.”

(Lee *et al.*, 2012)

Positionality defined

One’s social location or position assigned and negotiated as a result of combining various social factors or identifiers; including but not limited to:

> race, sex, class, gender, ability, age, religion, sexual orientation, nationality, physical stature, education, occupation, relational status, language.

Hearn, “Positionality, Intersectionality and Power,” 42

Breakout 4:
Power & Positionality in Facilitation

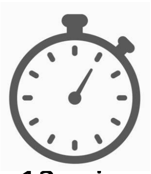

12 min

- Spend 5 minutes writing on your paper how issues of power and positionality may impact your facilitation from your perspective
- Consider issues of status, age, gender, and others that are important in your context
- Share with a neighbor

Multipartiality

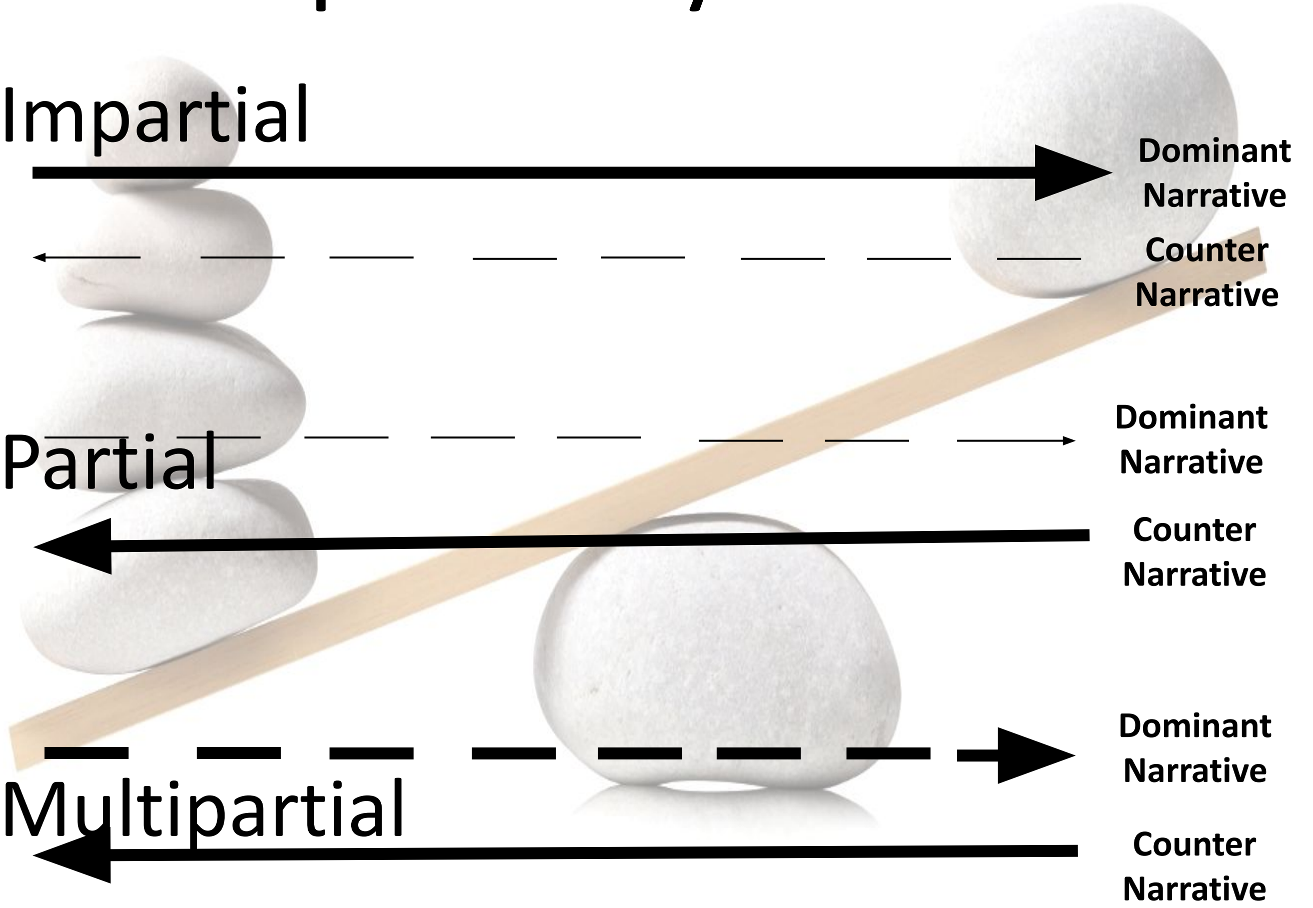

Breakout 5: Multipartial Facilitation in Practice

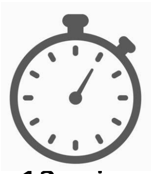

12 min

- Spend 5 minutes reading the page on multipartial facilitation.
- What questions do you have? Write them in the space below for notes.
- Now, with your colleagues, identify situations real or imagined (if they really happened, please anonymize the participants) when you could apply multipartial facilitation.

Part 2 of pre-work: Roles of Facilitators

Make it safe: Take time to tell/show the group members that the workshop is a safe place to be honest about their ideas and feelings. Everyone’s ideas are worth hearing.

Keep it constructive and positive: Remind members of your group to keep things positive and constructive. Ask the group how they want to deal with negativity and pointless venting. Remind them that the seminar is about working together to learn, not complaining about their current situation or discounting the ideas of others in the interest of a personal agenda.

Make the discussion functional: At the start of each session, explain the goals of the session to the group. Try to keep the group on task without rushing them. If the conversation begins to move beyond the main topic, bring the discussion back to the main theme of the session.

Give members of the group functional roles and responsibilities: Assign or ask for volunteers to take notes, keep track of time, and report out in the larger group at the end of the session. Functional roles help keep participants engaged.

Give all participants a voice: In a group, there are likely to be issues of intimidation and power dynamics that can play out in ways that allow certain members of the group to dominate and others to remain silent. At the start of the conversation, mention that the group is mixed by design, and point out that a diversity of perspectives is an essential part of the process. Remind group members to respect all levels of experience. It’s important that everyone’s voice is heard!

Facilitating Small-group Learning

Principles of Backward Design

1. Define learning goals / outcomes
2. Describe main small-group activity
3. Front-end: What information / prompts will participants need?
4. Back-end: What points are key for wrap up?
5. Synthesis and connections to next section

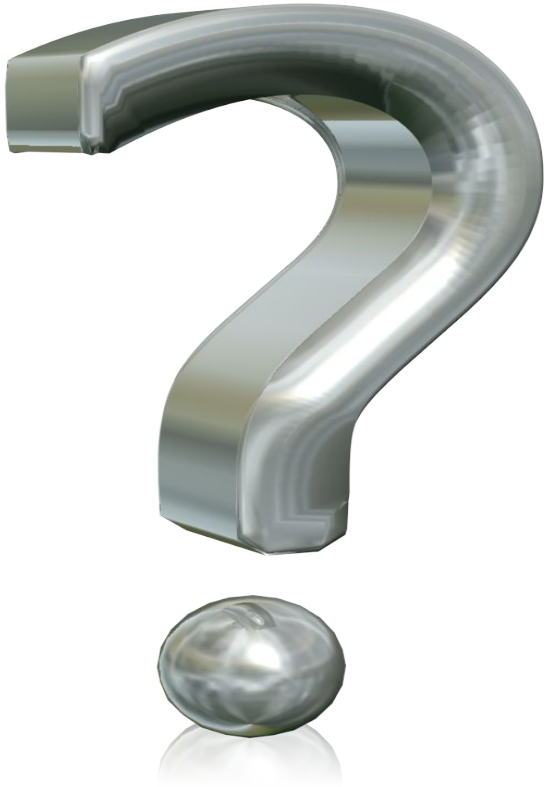

TZ/NG Facilitator Training

Bennett Goldberg, PhD
Professor of Physics and Astronomy
Faculty Director, Program Evaluation Core
Former Director, Searle Center for Advancing Learning and Teaching

Day 3/4, August 24, 2022

Northwestern University

University of Abuja

Dr. Fatima Kyari & Dr. Rifkatu Mshelia-Reng

Muhimbili University of Health and Allied Sciences

Dr. Emmanuel Balandya & Dr. Deodatus Kakoko

University of Ilorin

University of Ibadan

Catholic University of Health and Allied Sciences

Kilimanjaro Christian Medical University College

We Learn Together

We are here together in the same rooms to learn from each other. The wisdom is in the room.

We seek to explore new ideas, practice together, role play, in the process of developing facilitation skills.

We are aware of and acknowledge the power and positionality differential, and seek that such differential is, when necessary, overcome to enhance learning.

Guidelines for dialogue and participation

- Step up, step back
- Speak from your own experiences
- Challenge ideas, not people
- Consider and acknowledge impact as well as intent

Mute microphone if not speaking

Feel free to turn on video

Use the chat for questions or comments

“Raise hand” if you want to unmute

Methods

Modeling

- Demonstration
- Observation
- Experience
- Practical

Reflection

- Understanding
- Frameworks
- Context
- Generalize

Practice

- Application
- Repetition
- Assessment

Breakout 7 - Backward Design #1

As a facilitator…

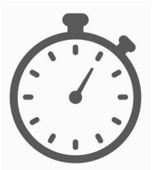

10 min

How is backward design different from traditionally planning of teaching or facilitation?

As a facilitator using backward design, how is your mindset different?

[Notes here]

My backward design process…

**Learning Goals** Identify desired results	**Evidence of learning -** Determine acceptable evidence	**Learning experiences & activities**	**Key facilitation actions**

My backward design process…

Learning Goals	Evidence of learning -	Learning experiences & activities	Key facilitation actions
1. Describe structure and purpose of backward design process<br>2. Apply backward design process in small-group learning			

My backward design process…

Learning Goals	Evidence of learning -	Learning experiences & activities	Key facilitation actions
1. Describe structure and purpose of backward design process<br>2. Apply backward design process in small-group learning	1. Participants construct key steps for themselves, adapt with feedback<br>2. Participants provide each other accurate & constructive feedback		

My backward design process…

**Learning Goals**	**Evidence of learning -**	**Learning experiences & activities**	**Key facilitation actions**
1. Describe structure and purpose of backward design process<br>2. Apply backward design process in small-group learning	1. Participants construct key steps for themselves, adapt with feedback<br>2. Participants provide each other accurate & constructive feedback	1. Breakout on backward design model; group report<br>2. Recap backward design<br>3. Personal time writing in table<br>4. Feedback process<br>5. Breakout sharing ideas and getting feedback.	

My backward design process…

Learning Goals	Evidence of learning -	Learning experiences & activities	Key facilitation actions
1. Describe structure and purpose of backward design process<br>2. Apply backward design process in small-group learning	1. Participants construct key steps for themselves, adapt with feedback<br>2. Participants provide each other accurate & constructive feedback	1. Breakout on backward design model; group report<br>2. Recap backward design<br>3. Personal time writing in table<br>4. Feedback process<br>5. Breakout sharing ideas and getting feedback.	1. Lead with prompt<br>2. Note progress & areas of uncertainty<br>3. Give sufficient time, challenge for advanced participants<br>4. Feedback on feedback process

Questions/Reflections on pre-work

- Where have you used backward design?
- What are your struggles with it?
- What are your questions?

Your time for backward design

5 min of individual work time to add your pre-work ideas to the same table here.

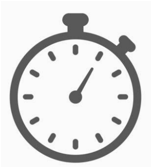

5 min

Feedback - SBIC

S - Situation; describe what you observed, be specific as possible about the actions, words, gestures used.

B - Behavior; what about the behavior was effective; what about the behavior was ineffective

I - Impact; what was the impact on participants

C - Challenge; what can be done more, to have greater impact, what should be shifted

Be positive, supportive, clear, about the actions…

Breakout 8 - Backward Design #2

15 min

- 10 min of group time to share your backward design example in the table here.
- Each person present; one or two with brief feedback
- Go around and take notes on your pad

Roles of Facilitators

Make it safe: Take time to tell/show the group members that the workshop is a safe place to be honest about their ideas and feelings. Everyone's ideas are worth hearing.

Keep it constructive and positive: Remind members of your group to keep things positive and constructive. Ask the group how they want to deal with negativity and pointless venting. Remind them that the seminar is about working together to learn, not complaining about their current situation or discounting the ideas of others in the interest of a personal agenda.

Make the discussion functional: At the start of each session, explain the goals of the session to the group. Try to keep the group on task without rushing them. If the conversation begins to move beyond the main topic, bring the discussion back to the main theme of the session.

Give members of the group functional roles and responsibilities: Assign or ask for volunteers to take notes, keep track of time, and report out in the larger group at the end of the session. Functional roles help keep participants engaged.

Give all participants a voice: In a group, there are likely to be issues of intimidation and power dynamics that can play out in ways that allow certain members of the group to dominate and others to remain silent. At the start of the conversation, mention that the group is mixed by design, and point out that a diversity of perspectives is an essential part of the process. Remind group members to respect all levels of experience. It's important that everyone's voice is heard!

Facilitating Small-group Learning

Principles of Backward Design

1. Define learning goals / outcomes
2. Describe main small-group activity
3. Front-end: What information / prompts will participants need?
4. Back-end: What points are key for wrap up?
5. Synthesis and connections to next section

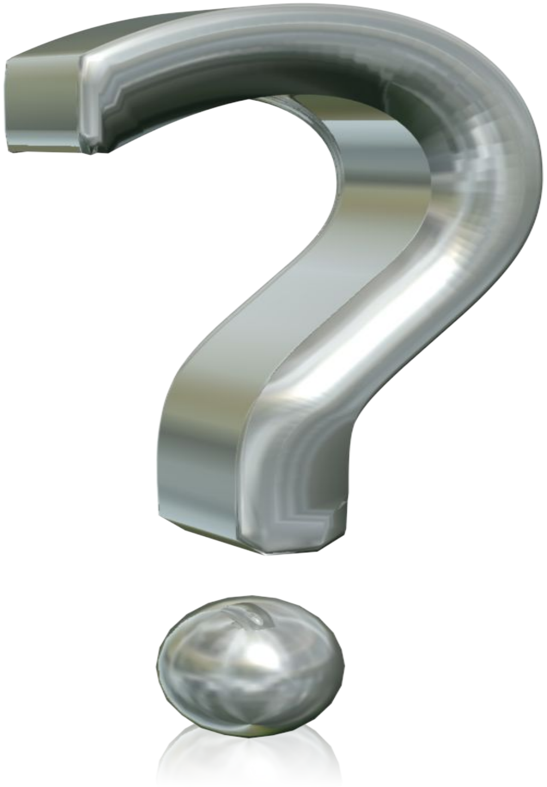

TZ/NG Facilitator Training

Bennett Goldberg, PhD
Professor of Physics and Astronomy
Faculty Director, Program Evaluation Core
Former Director, Searle Center for Advancing Learning and Teaching

Day 4/4, August 31, 2022

Northwestern University

University of Abuja

Dr. Fatima Kyari & Dr. Rifkatu Mshelia-Reng

Muhimbili University of Health and Allied Sciences

Dr. Emmanuel Balandya & Dr. Deodatus Kakoko

University of Ilorin

University of Ibadan

Catholic University of Health and Allied Sciences

Kilimanjaro Christian Medical University College

We Learn Together

We are here together in the same rooms to learn from each other. The wisdom is in the room.

We seek to explore new ideas, practice together, role play, in the process of developing facilitation skills.

We are aware of and acknowledge the power and positionality differential, and seek that such differential is, when necessary, overcome to enhance learning.

Guidelines for dialogue and participation

- Step up, step back
- Speak from your own experiences
- Challenge ideas, not people
- Consider and acknowledge impact as well as intent

Mute microphone if not speaking

Feel free to turn on video

Use the chat for questions or comments

“Raise hand” if you want to unmute

Methods

Modeling

- Demonstration
- Observation
- Experience
- Practical

Reflection

- Understanding
- Frameworks
- Context
- Generalize

Practice

- Application
- Repetition
- Assessment

Breakout 9 - Share Backward Designs

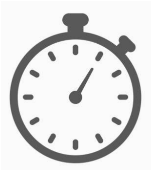

10 min

Backward Design Worksheet

- Meet in groups of 3;
- Each present briefly their design (2 min)
- Feedback based on questions (2 min)

Feedback on backward design

**Learning Goals** Identify desired results	**Evidence of learning - Determine** acceptable evidence	**Learning experiences & activities**	**Key facilitation actions**
Are they student-centered? Do they identify a skill, a competency, a measurable outcome?	Is the evidence aligned? Is the evidence direct (work product) or indirect (self-report of perception)? What is the level of evidence in Bloom's Taxonomy?	Are they active-learning activities? Will participants be engaged? Do they think and create for themselves? How are they aligned with goals and measures?	Do the actions have key observations? Are the observations student-centered? Are the results of the observations actionable in the moment? Is there reflection and learning?

My backward design process…

Learning Goals	Evidence of learning -	Learning experiences & activities	Key facilitation actions
1. Describe structure and purpose of backward design process<br>2. Apply backward design process in small-group learning	1. Participants construct key steps for themselves, adapt with feedback<br>2. Participants provide each other accurate & constructive feedback	1. Breakout on backward design model; group report<br>2. Recap backward design<br>3. Personal time writing in table<br>4. Feedback process<br>5. Breakout sharing ideas and getting feedback.	1. Lead with prompt<br>2. Note progress & areas of uncertainty<br>3. Give sufficient time, challenge for advanced participants<br>4. Feedback on feedback process

Questions/Reflections on pre-work

- Where have you used backward design?
- What are your struggles with it?
- What are your questions?

Build a run-of-show together

- Run-of-show --
- Why do we do it?
- What should it contain?
- Why is it focused on facilitation?

Facilitating Small-group Learning

Principles of Backward Design

1. Define learning goals / outcomes
2. Describe main small-group activity
3. Front-end: What information / prompts will participants need?
4. Back-end: What points are key for wrap up?
5. Synthesis and connections to next section

Roles of Facilitators

Make it safe: Take time to tell/show the group members that the workshop is a safe place to be honest about their ideas and feelings. Everyone's ideas are worth hearing.

Keep it constructive and positive: Remind members of your group to keep things positive and constructive. Ask the group how they want to deal with negativity and pointless venting. Remind them that the seminar is about working together to learn, not complaining about their current situation or discounting the ideas of others in the interest of a personal agenda.

Make the discussion functional: At the start of each session, explain the goals of the session to the group. Try to keep the group on task without rushing them. If the conversation begins to move beyond the main topic, bring the discussion back to the main theme of the session.

Give members of the group functional roles and responsibilities: Assign or ask for volunteers to take notes, keep track of time, and report out in the larger group at the end of the session. Functional roles help keep participants engaged.

Give all participants a voice: In a group, there are likely to be issues of intimidation and power dynamics that can play out in ways that allow certain members of the group to dominate and others to remain silent. At the start of the conversation, mention that the group is mixed by design, and point out that a diversity of perspectives is an essential part of the process. Remind group members to respect all levels of experience. It's important that everyone's voice is heard!

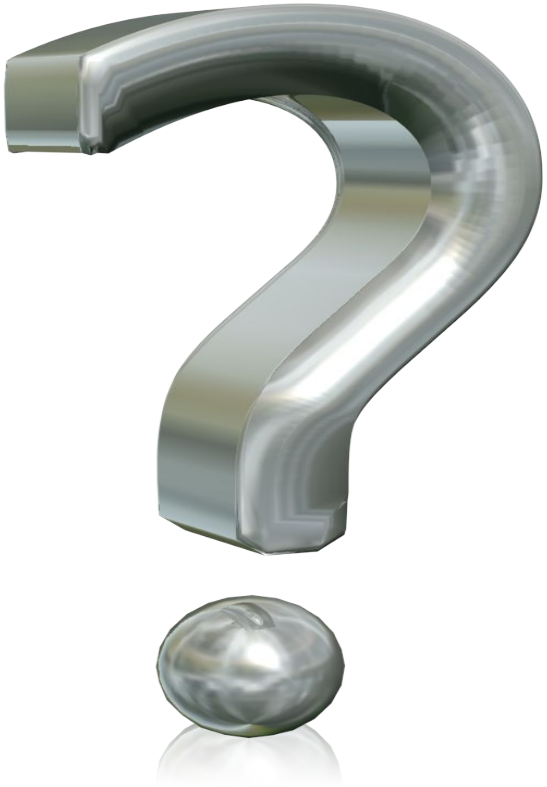

TZ/NG Facilitator Training

Bennett Goldberg, PhD
Professor of Physics and Astronomy
Faculty Director, Program Evaluation Core
Former Director, Searle Center for Advancing Learning and Teaching

Day 5/4, September 7, 2022

Northwestern University

University of Abuja

Dr. Fatima Kyari & Dr. Rifkatu Mshelia-Reng

Muhimbili University of Health and Allied Sciences

Dr. Emmanuel Balandya & Dr. Deodatus Kakoko

University of Ilorin

University of Ibadan

Catholic University of Health and Allied Sciences

Kilimanjaro Christian Medical University College

We Learn Together

We are here together in the same rooms to learn from each other. The wisdom is in the room.

We seek to explore new ideas, practice together, role play, in the process of developing facilitation skills.

We are aware of and acknowledge the power and positionality differential, and seek that such differential is, when necessary, overcome to enhance learning.

Guidelines for dialogue and participation

- Step up, step back
- Speak from your own experiences
- Challenge ideas, not people
- Consider and acknowledge impact as well as intent

Mute microphone if not speaking

Feel free to turn on video

Use the chat for questions or comments

“Raise hand” if you want to unmute

Methods

Modeling

- Demonstration
- Observation
- Experience
- Practical

Reflection

- Understanding
- Frameworks
- Context
- Generalize

Practice

- Application
- Repetition
- Assessment

Run-of-show

**Facilitator**	**Timing**	**Slides**	**Activity/topic**
	0-5		Folks arrive. Set up and Introductions, Goals of the session; session norms; Overall framing of the project;
	5-10		Prompt; initial work; recap or new material overview
	10-25		Activity 1: Breakout at tables; group report and synthesis
	25-35		Setup second major learning goal; Detailed look at our evaluation and cycles of improvement; Prompt
	35-45		Activity 2: In pairs, turn to your neighbor for think-pair-share: Share a one-pager of the example of feedback from…
	45-55		Questions and next steps
Total	55		

Build a run-of-show

- Run-of-show --
- Why do we do it?
- What should it contain?
- How is it focused on facilitation?

Breakout #10 - Run-of-show

- Why do we do it?
- What should it contain?
- How is it focused on facilitation?

Notes here

Facilitating Small-group Learning

Applying principles of Backward Design

1. Define learning goals / outcomes
2. Describe main small-group activity
3. Front-end: What information / prompts will participants need?
4. Back-end: What points are key for wrap up?
5. Synthesis and connections to next section

Dynamics of Facilitation

Make it safe:

Keep it constructive and positive:

Give members of the group functional roles and responsibilities:

Give all participants a voice:

Support all learners to learn and be engaged:

Support the exchange of ideas:

Check whether learning is happening among students:

Listen and summarize:

Functional discussions:

Roles of Facilitators 1

Make it safe: Take time to tell/show the group members that the workshop is a safe place to be honest about their ideas and feelings. Everyone's ideas are worth hearing.

Keep it constructive and positive: Remind members of your group to keep things positive and constructive. Ask the group how they want to deal with negativity and pointless venting. Remind them that the seminar is about working together to learn, not complaining about their current situation or discounting the ideas of others in the interest of a personal agenda.

Roles of Facilitators 2

Make the discussion functional: At the start of each session, explain the goals of the session to the group. Try to keep the group on task without rushing them. If the conversation begins to move beyond the main topic, bring the discussion back to the main theme of the session.

Give members of the group functional roles and responsibilities: Assign or ask for volunteers to take notes, keep track of time, and report out in the larger group at the end of the session. Functional roles help keep participants engaged.

Give all participants a voice: In a group, there are likely to be issues of intimidation and power dynamics that can play out in ways that allow certain members of the group to dominate and others to remain silent. At the start of the conversation, mention that the group is mixed by design, and point out that a diversity of perspectives is an essential part of the process. Remind group members to respect all levels of experience. It’s important that everyone’s voice is heard!

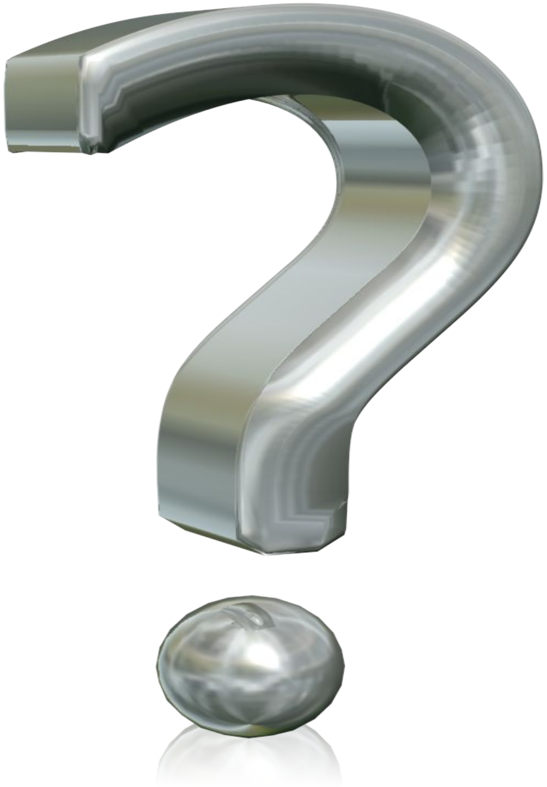

Supplement: Supplementary material Training Slides [file NIHMS1994830-supplement-Supplementary_material_Training_Slides.pdf]
