# Supplementary material for: Training in the art and science of facilitation to scale research mentor training in low and middle income countries
Source: Front Educ (Lausanne). Author manuscript; Available in PMC 2024 Jun 6. (PMC11155035; doi:10.3389/feduc.2023.1270480)
Supplement: Supplementary Material Survey [file NIHMS1994830-supplement-Supplementary_Material_Survey.pdf]

Tools Saved at 8:56 AM Draft Preview Publish

TZ/NG Facilitation Training Eval Sept 2022

ExpertReview score Fair

Block 0 - Description

Q1

Dear Colleagues,

Thank you so much for participating in the TZ/NG Facilitation Training!

Please complete this brief survey so that we can understand the impact of the training and improve it for future offerings. The survey is anonymous and optional. At the end, you are asked whether you would be available for an interview. Any name or email provided will be stripped from the data set prior to analysis.

Please contact me, Bennett Goldberg, with any questions.

Thanks again!

Import from library Add new question

Add Block

Block 1 - Knowledge, skill and confidence advancement

Q2

To what extent did the training advance your knowledge of....

	None at all	A little	A moderate amount	A lot	A great deal
Facilitation	○	○	○	○	○
Active learning	○	○	○	○	○
Backward design	○	○	○	○	○
Small group learning interactions	○	○	○	○	○
observing participant interactions in groups	○	○	○	○	○

Q3

To what extent did the training advance your ability to design...

	None at all	A little	A moderate amount	A lot	A great deal
A facilitation session in a workshop	◯	◯	◯	◯	◯
An active learning activity	◯	◯	◯	◯	◯
A facilitation session using backward design	◯	◯	◯	◯	◯
A small group learning interaction	◯	◯	◯	◯	◯
A list of key questions for observing participant interactions in groups	◯	◯	◯	◯	◯

Q4

How much confidence have you gained in the following

	None	A little	A moderate amount	A great deal
facilitating small group learning interactions	◯	◯	◯	◯
Instructing using active learning approaches	◯	◯	◯	◯
designing instruction and/or facilitation using backward design	◯	◯	◯	◯
Running a small group learning interaction	◯	◯	◯	◯
Observing participants and shifting your facilitation in response to what you observe	◯	◯	◯	◯

Import from library

Add new question

Add Block

Block 2 - CIQ

Q5

Describe when you were *most* engaged and why during the training sessions

Q6

Describe when you were *least* engaged and why during the training sessions

Import from library Add new question

Add Block

Block 3 - Takeaways and Key Applications to Practice

Q12

Major takeaways - what are the top three key aspects of facilitating small group interactions that were most important to you

Major Takeaway #1

Major Takeaway #2

Major Takeaway #3

Q13

What top three practices that you learned will you be implementing in your facilitation and/or teaching? Please describe the practice ***and*** how you will implement it.

Top practice #1

Top practice #2

Top practice #3

Import from library Add new question

Add Block

Block 4 - Attendance; Demographics; Career Stage

Q20

We held five sessions. Please choose the number of sessions you participated in

- ◯ 1 - 2 Sessions
- ◯ 3 - 4 Sessions
- ◯ All 5 Sessions

Q21

Please write your title (or titles) and current position

Q22

Please describe your current career stage

Q23

Please indicate how many years you have been teaching

- ◯ Have not yet taught
- ◯ 1-4 years of teaching experience
- ◯ 5-9 years of teaching experience
- ◯ 10+ years of teaching experience

Q24

What is your current gender identity? Please select one or use the write-in option.

- ◯ Female
- ◯ Male
- ◯ Non-binary / third gender
- ◯ Prefer not to say
- ◯ Write-in option

Import from library Add new question

Add Block

Block 5 - Willingness to be interviewed

Q14

We are seeking to interview participants as part of our evaluation.

The interview will be one-on-one with an individual not involved the training. The information will be recorded and transcribed, and identifying information will be removed prior to analysis. Interviews will take place later in September and October. If you are willing to be interviewed, please provide your name and preferred email address below. This is entirely optional.

Name

Preferred email address

Import from library

Add new question

Add Block

End of Survey

We thank you for your time spent taking this survey.

Your response has been recorded.
